# Yin Yang 1 Intronic Binding Sequences and Splicing Elicit Intron-Mediated Enhancement of Ubiquitin C Gene Expression

**DOI:** 10.1371/journal.pone.0065932

**Published:** 2013-06-12

**Authors:** Marzia Bianchi, Rita Crinelli, Elisa Giacomini, Elisa Carloni, Lucia Radici, Mauro Magnani

**Affiliations:** Department of Biomolecular Sciences, Biochemistry and Molecular Biology Section, University of Urbino “*Carlo Bo*”, Urbino, Italy; International Centre for Genetic Engineering and Biotechnology, Italy

## Abstract

In a number of organisms, introns affect expression of the gene in which they are contained. Our previous studies revealed that the 5′-UTR intron of human ubiquitin C (*UbC*) gene is responsible for the boost of reporter gene expression and is able to bind, *in vitro*, Yin Yang 1 (YY1) *trans*-acting factor. In this work, we demonstrate that intact YY1 binding sequences are required for maximal promoter activity and YY1 silencing causes downregulation of luciferase mRNA levels. However, YY1 motifs fail to enhance gene expression when the intron is moved upstream of the proximal promoter, excluding the typical enhancer hypothesis and supporting a context-dependent action, like intron-mediated enhancement (IME). Yet, almost no expression is seen in the construct containing an unspliceable version of *UbC* intron, indicating that splicing is essential for promoter activity. Moreover, mutagenesis of YY1 binding sites and YY1 knockdown negatively affect *UbC* intron removal from both endogenous and reporter transcripts. Modulation of splicing efficiency by YY1 *cis*-elements and protein factor may thus be part of the mechanism(s) by which YY1 controls *UbC* promoter activity. Our data highlight the first evidence of the involvement of a sequence-specific DNA binding factor in IME.

## Introduction

Ubiquitin (Ub), in the context of the ubiquitin proteasome system (UPS) is responsible for much of the regulated proteolysis in the cell, but has non-degradative functions as well: it is in fact involved in the control of many cellular activities, ranging from signal transduction to transcription, from endocytosis to protein trafficking, from DNA repair to cell survival and proliferation [1]. Ubiquitin is a versatile 76 amino-acid polypeptide that can be reversibly attached to other proteins and lies at the core of an elaborate post-translational modification pathway [2]. The peculiar features of ubiquitin at the function level are also displayed at the gene level. Ubiquitin is typically synthesized as a fusion protein cotranslationally processed in the mature form, and in mammals is obtained starting from four genes [3]–[6].

The *UbC* gene, coding for polyubiquitin, is the most studied and characterized because of its inducibility in response to various cell challenges [7]. Moreover, the *UbC* promoter is a widely used regulatory sequence to drive a high and sustained level of transgene expression [8]–[10].

A feature shared by different polyubiquitin genes, at least in plants, is the presence of introns which are commonly located within the 5′ untranslated region (5′-UTR): introns in this position were reported to mediate high gene expression, by a mechanism termed intron-mediated enhancement (IME). IME was demonstrated for rice [11], Arabidopsis and several other multicellular plants [12]. The ability of introns to stimulate gene expression is a largely stated matter in a wide range of organisms, including mammals, nematodes, insects, fungi and plants [13]. The two general ways by which introns enhance mRNA level and/or translational competence is by the process of IME, not yet completely defined, or by acting as transcriptional enhancer or alternative promoter, depending on *cis*-elements located within the intron spanning sequence [13]. Diagnostic tests to discriminate enhancers from IME are based on whether the intron can still enhance expression if it is moved outside of the transcribed sequence or its orientation is changed; whether it relies on specific *cis*-elements (other than consensus splice sites) to which *trans*-acting factors bind; and, finally, whether the intron is able to stimulate gene expression with a minimal or no promoter. Regardless of the mechanism involved, both intron-dependent effects add a further layer of regulation of gene expression, besides the mostly described alternative promoters, alternative splicing, mRNA stability control, and so on [14].

The potential intron engineering as a means of enhancing transgene expression has been exploited in plants and the molecular basis of the enhanced expression was examined as well [15]. The development of ubiquitin-based expression vectors concerns mammalian polyubiquitin gene promoters (mainly *UbC* promoter), which are widely employed as a tool for gene delivery [10]. *UbC*-based expression vectors combined several positive features such as constitutive high-level transgene expression, and increased persistence after a single administration, which make them more robust than other routinely employed viral promoters, regardless of the intron inclusion in the transcription promoter region [16]. On the other hand, the human ubiquitin *C* gene has been investigated as concerns its upregulation upon cell challenge with different stressors and also for its contribution to the maintenance of ubiquitin homeostasis [17], avoiding or opposing perturbations in ubiquitin intracellular levels, in both physiological and pathological conditions [18]. However, to the best of our knowledge, there are no detailed studies on *UbC* promoter at the molecular level and the complete characterization of the molecular mechanisms underlying polyubiquitin *C* expression, in humans, remains an open question.

In our previous work, we addressed the promoter study and found that all the regulatory elements required, *in vivo*, for a sustained reporter gene expression are present within a ∼1.25-kb genomic fragment that contains 371 nucleotides upstream of the transcription start point (herein referred to as proximal promoter) and 876 nucleotides of downstream untranslated sequence, which includes the unique 812 nt-intron region of the gene. Intron removal, or replacement with a heterologous chimeric intron, caused a drastic drop of promoter activity [19]. In the present work we set out to investigate the molecular mechanism(s) by which the 5′-UTR *UbC* intron enhances gene expression; in particular we tested whether the *cis*-elements, able to bind *in vitro* the ubiquitous Sp1 and YY1 transcription factors, were involved in the stimulation of reporter gene transcription. Interestingly, and contrary to expectations, our results unravel a novel mechanism of action for the intron which is reminiscent of the IME effect, although requiring the presence of the identified YY1 transcription factor binding motifs.

## Materials and Methods

### Promoter Constructs

**Figure 1:**
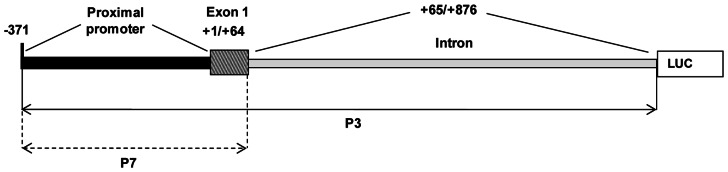
Schematic representation of the investigated human *UbC* promoter region. The diagram shows the *UbC* promoter region spanning from nt −371 (with respect to the transcription start site) to nt +876 cloned in the P3 reporter construct, including the proximal promoter (PP), the first exon and the unique intron of the 5′-UTR of the gene. The promoter fragment devoid of the intron (nt −371/+64), cloned into the P7 vector, is also highlighted.

Cloning of the regulatory and partial transcribed regions of *UbC* gene was previously described [19]. Construct P3 contains 371 nt upstream to the transcription start (herein referred to as proximal promoter, PP) and the 5′-UTR region of *UbC* gene, composed of the 63-nt exon 1 and the 812-nt unique intron. Construct P7 is similar to P3, except that it lacks the 5′-UTR intron (Figure 1).

New reporter vectors were generated starting from the P3 plasmid. Constructs Int(s)-PP-Ex1 and Int(as)-PP-Ex1 were created by amplification of the wild-type P3 with the following primer pair engineered to be cut with Sac I: 5′-GTGATCGGAGCTCGGTGAGTAGCGGGCTGCTGGG-3′ and 5′-ATCTGCGAGCTCTAACAAAAAAGCCAAAAACGGCC-3′. The PCR product, including the *UbC* intron, was cloned in the construct P7, upstream of the proximal promoter region, in both sense and antisense direction.

Constructs [Ex1-Int](s)-PP and [Ex1-Int](as)-PP, where both exon 1 and flanking intron were positioned upstream of the PP region, in sense or antisense orientation, were generated by amplifying P3 with the forward primer 5′-GTGGCAGAGCTCGTTCCGTCGCAGCCGGGATTTG-3′ bearing a Sac I cutting sequence and the same reverse primer reported above. The reporter construct carrying the −371/−38 nt deletion was obtained from the P3 reporter vector amplified with the primer −37 forward 5′-GGTACCGAGCTCGATGATTATATAAGGACGCG-3′, bearing a Sac I cutting sequence, and the primer +876 reverse 5′-TGGAAGCTTGTCTAACAAAAAAGCCAAAAACGGC-3′, bearing a Hind III recognition site. The Sac I-Hind III cut PCR product was cloned into pGL3-basic, generating the N-terminal truncated construct P3Δ(−371/−38).

All the constructs were confirmed by sequence analysis using a PE 310 Perkin Elmer capillary sequencer.

### Site-directed Mutagenesis

The QuikChange Site-Directed Mutagenesis kit or the QuikChange Lightning Multi Site-Directed Mutagenesis kit (Stratagene, La Jolla, CA, USA) were used according to the manufacturer’s instructions for single-site or multi-site mutations of transcription factor binding motifs, respectively. Mutations in Sp1 binding sequences were as follows: sites a-d were changed from GGGNGG to ACANGG. YY1 intronic binding motifs (e–f) were changed from ATGGCGG to AGTGCAC, while the upstream YY1 binding site (g), located in the proximal promoter, was changed from GGACATT to GTAAGCT. Primers used for mutagenesis reactions are listed in Table 1. In each case nucleotides reported to be critical for factor binding were changed. Mutagenesis of 5′- and 3′-splice site consensus sequences was performed using the QuikChange Lightning Multi Site-Directed Mutagenesis kit and the primers shown in Table 1.

**Table 1:** Oligonucleotides used for EMSA and site-directed mutagenesis.

NAME (position)	SEQUENCES (5′ to 3′)	WILD-TYPE BINDING SEQUENCE
**Sp1 mutagenesis**		Sp1 consensus
		g/t G/a GG C/a GG G/a G/a
Sp1-ODN 1 Fwd (+180)	GCCCTGAACTGGGGGTT**ACAGGG**AGCGCAGCAAAATGGC	**GGGGGG**
Sp1-ODN 1 Rev (+218)	GCCATTTTGCTGCGCTCCC**TGT**AACCCCCAGTTCAGGGC	
Sp1-ODN 3 Fwd (+418)	GGTTTGTCGTCTGTTGCGG**ACACGG**CAGTTATGGCGGTGC	**GGGCGG**
Sp1-ODN 3 Rev (+457)	GCACCGCCATAACTGCCG**TGT**CCGCAACAGACGACAAACC	
Sp1-ODN IVb Fwd (+524)	GTTGGCTTATAATGCA**ACATGG**GGCCACCTGCCGGTAGG	**GGGTGG**
Sp1-ODN IVb Rev (+562)	CCTACCGGCAGGTGGCCCCA**TGT**TGCATTATAAGCCAAC	
Sp1-ODN 5 Fwd (+635)	GCCGGACCTCTGGTGAG**ACAAGG**GATAAGTGAGGCGTC	**GGGAGG**
Sp1-ODN 5 Rev (+672)	GACGCCTCACTTATCCCT**TGT**CTCACCAGAGGTCCGGC	
**YY1 mutagenesis**		YY1 consensus(1)C**GCCAT**nTTAAn**ATGGC**G
YY1-ODN IIa Fwd (+198)	GGGGGAGCGCAGCAAAA**GT**GC**AC**CTGTTCCCGAGTCTTG	AAa**ATGGC** G
YY1-ODN IIa Rev (+236)	CAAGACTCGGGAACAG**GTGCAC**TTTTGCTGCGCTCCCCC	C **GCCAT**tTT
YY1-ODN 3 Fwd (+435)	GGGGGCGGCAGTTA**GT**GC**AC**TGCCGTTGGGCAGTG	GTt**ATGGC** G
YY1-ODN 3 Rev (+469)	CACTGCCCAACGGCA**GTGCAC**TAACTGCCGCCCCC	C **GCCAT**aAC
YY1-ODN PP Fwd (−181)	CCCAGTATCAGCAGAAG**TAAGC**TTTAGGACGGGACTTGGG	G**GACAT**tTT
YY1-ODN PP Rev (−142)	CCCAAGTCCCGTCCTAAA**GCTTA**CTTCTGCTGATACTGGG	AAa**ATGTC**C
**Splice site mutagenesis**		**Wild-type splice sites**
5′ Splice site mut Fwd (+48)	*gctgtgatcgtcactcc* AGCCGTAGCGGGCTGCTGGGCTG	*tg* GTGA
3′ Splice site mut Fwd (+855)	CCGTTTTTGGCTTTTTTGTGGC*t* *caagcttggcattccgg*	TAG***a***
**EMSA**		
ODN 1 (+158/+212)	GTCTGGGTCCGCGAGCAAGGTTGCCCTGAACTGGGGGTTGGGGGGAGCGCAGCAA	
ODN 1a (+158/+185)	GTCTGGGTCCGCGAGCAAGGTTGCCCTG	
ODN 1b (+172/+199)	GCAAGGTTGCCCTGAACTGGGGGTTGGG	
ODN 1c (+186/+212)	AACTGGGGGTT**GGGGGG**AGCGCAGCAA	
ODN 1c mut (+186/+212)	AACTGGGGGTT**ACAGGG**AGCGCAGCAA	
ODN 5 (+645/+679)	TGGTGAGGGGAGGGATAAGTGAGGCGTCAGTTTCT	
ODN 5a (+645/+669)	TGGTGAG**GGGAGG**GATAAGTGAGGC	
ODN 5a mut (+645/+669)	TGGTGAG**ACAAGG**GATAAGTGAGGC	
ODN 5b (+655/+679)	AGGGATAAGTGAGGCGTCAGTTTCT	
ODN IVa (+477/+516)	ACCTTTGGGAGCGCGCGCCCTCGTCGTGTCGTGACGTCAC	
ODN 4 (+497/+526)	TCGTCGTGTCGTGACGTCACCCGTTCTGTT	
ODN IVb (+526/+565)	TGGCTTATAATGCA**GGGTGG**GGCCACCTGCCGGTAGGTGT	
ODN IVb mut(+526/+565)	TGGCTTATAATGCA**ACATGG**GGCCACCTGCCGGTAGGTGT	
ODN IVc (+547/+586)	GCCACCTGCCGGTAGGTGTGCGGTAGGCTTTTCTCCGTC	
ODN IVd (+577/+607)	TTCTCCGTCGCAGGACGCAGGGTTCGGGCCT	
ODN IIa (+208/+234)	AGCAA**AATGGCGGC**TGTTCCCGAGTCT	
ODN IIa mut (+208/+234)	AGCAA**AAGTGCACC**TGTTCCCGAGTCT	
ODN IIb (+235/+262)	TGAATGGAAGACGCTTGTGAGGCGGGCT	
ODN IIc (+263/+290)	GTGAGGTCGTTGAAACAAGGTGGGGGGC	

Fwd, forward; Rev, reverse; n, every base; PP, proximal promoter.

The numbers in brackets refer to the transcription start site of *UbC* gene identified as +1. The mutagenized Sp1 and YY1 binding sequences are in bold letters and nucleotide changes respect to the wild-type sequence are underlined. Sp1 and YY1 consensus sequences are also indicated in the third column. The italicized lower case letters in the primers used for splice site mutagenesis indicate the upper and downstream exon flanking sequences.

(1) YY1 binding motifs in the *UbC* intron are in the minus (antisense) strand; the one in the upstream proximal promoter (PP) sequence is in the sense strand.

The mutated sequence was verified by automated sequencing in both directions. Mutagenesis was performed on reporter construct P3 containing the −371/+876 fragment of *UbC* promoter region. The P3Δ and P7 plasmids carrying mutations in the harbored YY1 site(s) were obtained by PCR of the appropriate P3 mutagenized construct using the degenerate primer pairs previously reported (see above and ref 19, respectively).

### Cell Culture, Transfections, and RNA Extraction

Tissue culture media and supplements were purchased from Cambrex Biosciences. Cervical cancer cell line (HeLa), obtained from the American Type Culture Collection (ATCC), was maintained routinely in RPMI 1640 medium with 10% fetal bovine serum, 2 mM glutamine, 100 µg/ml streptomycin, and 100 U/ml penicillin at 37°C under 5% CO_2_. The day before transfection, cells were plated at a density of ∼3.5×10^5^ cells/well in 6-well plates. Transient transfections of plasmid DNA were performed with Effectene reagent (Qiagen Inc.), according to the manufacturer’s protocol.

For transfection of reporter constructs, 400 ng of DNA were added to each well. As a control for transfection efficiency, in some experiments 100 ng of a GFP expression plasmid were cotransfected with the wild-type (P3), mutant (YY1mut e–f) and intron-lacking (P7) reporter constructs. GFP expression, normalized to the housekeeping gene b2-microglobulin, was not statistically different among the cotransfected samples: 1.00±0.09 (P3), 1.16±0.08 (YY1mut e–f) and 0.91±0.12 (P7), respectively (n = 3). For luciferase/YY1 cotransfection experiments, cells were transfected with 400 ng of luciferase expressing plasmid and 50 ng of an expression vector for YY1 transcription factor. The YY1 expression construct was a gift from Prof. Salvatore Oliviero (University of Siena, Italy) and contains the full-length coding sequence of the human YY1, cloned in the pcDNA3 vector (Invitrogen) under control of the cytomegalovirus (CMV) promoter [20]. In a given experiment, the total amount of DNA was maintained constant by adding control vector.

Cells were harvested 48 h after transfection for RNA extraction or luciferase assay, unless otherwise specified. Total cellular RNA was prepared using RNeasy Plus Mini kit (Qiagen). To remove any trace of plasmid DNA, total RNA (up to 10 µg) was treated with 2 units of TURBO DNA-free (Ambion, Austin, TX) for 30 min at 37°C, according to the included protocol.

### Reverse Transcription (RT) and Quantitative Real-Time PCR (qRT-PCR)

TURBO-treated RNA (1 µg) was reverse-transcribed using the SuperScript® First-Strand Synthesis System (Invitrogen, Carlsbad, CA, USA), and oligo-dT (0.5 µg/reaction) or random hexamers (0.15 µg/reaction) as primers, in a final volume of 20 µl, essentially as indicated in the standard protocol.

cDNAs were used as templates in SYBR green quantitative RealTime PCR (qRT-PCR) assays, performed with the Hot-Rescue RealTime PCR kit (Diatheva s.r.l., Fano, Italy). PCR reactions were set up in a volume of 25 µl containing 1× Hot-Rescue RealTime Master Mix, 0.2 µM of gene specific primers, 0.625 units of Hot-Rescue DNA polymerase, 5 µl of a fifty-fold dilution of the RNase H-treated cDNA stock. DNA amplifications were carried out in 96-well reaction plates using ABI PRISM 7700 Sequence Detection System platform (Applied Biosystems, Foster City, CA). Each sample was analyzed in triplicate, and multiple blanks were included in each analysis. qRT-PCR primers (obtained from Sigma-Genosys Ltd, Haverhill, UK) were designed using Primer Express version 2.0.

Primer sequences, as well as the relative MgCl_2_ concentration and amplicon length were: luciferase (LUC), forward 5′-TGTACACGTTCGTCACATCTCATCT-3′ and reverse 5′-AGTGCAATTGTCTTGTCCCTATCG-3′ (3 mM MgCl_2_, 91 bp); UbC forward 5′-GTGTCTAAGTTTCCCCTTTTAAGG-3′ and reverse 5′-TTGGGAATGCAACAACTTTATTG-3′ (5 mM MgCl_2_, 76 bp); YY1, forward 5′-GAAGCCCTTTCAGTGCACGTT-3′ and reverse 5′-ACATAGGGCCTGTCTCCGGTAT-3′ (2.5 mM MgCl_2_, 102 bp); b2-microglobulin (B2M), forward 5′-GCCTGCCGTGTGAACCAT-3′ and reverse 5′-CATCTTCAAACCTCCATGATGCT-3′ (5 mM MgCl_2_, 91 bp); Green Fluorescent Protein (GFP), forward 5′-CCTGAAGTTCATCTGCACCA-3′ and reverse 5′-TGCTCAGGTAGTGGTTGTCG-3′ (3.5 mM MgCl_2_, 479 bp).

For the splicing efficiency studies the following primers were employed: intron probe II, forward 5′-GAGAGACCGCCAAGGGCTGTAG-3′ and reverse 5′-CGCATTAGCGAAGGCCTCAAG-3′ (3 mM MgCl_2_, 200 bp) to detect the endogenous unspliced *UbC* RNA; intron probe VI forward 5′-AGCTGAAGCTCCGGTTTTGAACTAT-3′ and LUC-1 reverse 5′-CATAGCCTTATGCAGTTGCTCTCCA-3′ (3.5 mM MgCl_2_, 292 bp) to detect the unspliced luciferase RNA driven by P3 and YY1mut e–f; pRL forward 5′-GGCAGGTAAGTATCAAGGTTACAAG-3′ and LUC-1 reverse reported above (4.5 mM MgCl_2_, 270 bp) to detect the unspliced luciferase RNA driven by P7+chimeric intron construct. Cycle conditions were 95°C for 10 min followed by 40 cycles of 15 s at 95°C, 15 s at 60°C and 30 s at 72°C. After the run, the melting curve of each amplicon was examined to determine the specificity of the product. Amplification plots were analyzed using SDS 1.9.1 software (Applied Biosystems) and relative expression data were calculated with the 2^−ΔΔ*C*^_T_ method [21].

For absolute quantification in qRT-PCR, plasmid DNA bearing the different targets, amplified and purified by standard procedures, was linearized and then quantified at 260 nm using the Nanodrop ND-1000 System (NanoDrop Technologies, Wilmington, DE). From each plasmid serial dilutions from 10^7^ to 10^1^ copies were prepared and used as standards in the RT-PCR assay.

### Luciferase Assay

Cell extracts were subjected to luciferase assay 48 h post-transfection using the Luciferase assay reagent (Promega), essentially as reported in [19]. Luciferase activity was determined on a FLUOstar OPTIMA multifunction microplate reader (BMG-LABTECH GmbH). The light intensities were normalized against total protein concentration, determined by the Bradford method (DC Protein Assay; BioRad) [22]. Promoter activity was expressed as a percentage of that measured for the wild-type construct (P3), which was set equal to 100%.

### siRNA Duplex Transfections

Predesigned siRNA duplexes against human YY1 (YY1_1, SI00051912 and YY1_3, SI00051926) and nonsilencing control siRNA Alexa Fluor 488 (AF 488; 1027284) were purchased from Qiagen. Transfections with si-YY1 were performed using the RiboCellin reagent (BioCellChallenge SAS, France), according to the manufacturer’s instructions. The final siRNA concentration was 25 nM. Forty-eight, 72 and 96 hours after transfection, cells were harvested and assayed for mRNA content and protein expression. Fluorescent control siRNA was used for monitoring the transfection efficiency, by means of fluorescence microscopy. For the luciferase reporter studies, cells treated with YY1 specific and control siRNAs were transfected 24 h thereafter with 400 ng of reporter vector, with Effectene. Cotransfected cells were assayed 48 h after reporter transfection for mRNA and protein expression.

### Assessment of Splicing

Following transfection of the constructs P3 (wild-type) and P3-SSmut (carrying mutations of both 5′- and 3′-splice site consensus motifs) in HeLa cells, total RNA was extracted and cDNA synthesized using random hexamers as primers. PCR was performed with Hot-Rescue DNA Polymerase (Diatheva), using 1 µl of reverse transcribed cDNA. The forward and reverse primers, bridging the intron, were derived from the first exon of the 5′-UTR (5′-TCTTGTTTGTGGATCGCTGTGATC-3′) and from the luciferase coding sequence (5′-AGTGCAATTGTCTTGTCCCTATCG-3′), respectively. The constructs P3 and P7 were used to generate PCR fragments that corresponded to the size of the unspliced (P3) and spliced (P7) transcripts. To exclude amplification from contaminating plasmid DNA, PCR was performed on RNA samples not reverse transcribed, as a negative control. Reactions were run in a Perkin-Elmer thermal cycler using the following conditions: 10 min at 95°C for 1 cycle, followed by 30 cycles of 15 sec at 95°C, 15 sec at 62°C and 1 min at 72°C. PCR products were fractionated by standard agarose gel electrophoresis.

### RNA Stability

For the actinomycin D experiment, HeLa cells were transfected with 400 ng of luciferase expressing plasmids (P3 and YY1mut e–f). Actinomycin D (SIGMA; 5 µg/ml final) was added to the medium 48 h post-transfection. At the times indicated, total RNA was extracted and analyzed by RealTime PCR with specific primers for the luciferase target gene and for b2-microglobulin, as the housekeeping control gene.

### Whole Cell Lysate Preparation and Western Blot Analysis

For immunoblotting, HeLa cells were harvested and lysed in 50 mM Tris-HCl, pH 7.8; 0.25 M sucrose, 2% (w/v) SDS, 10 mM N-ethylmaleimide (NEM) supplemented with fresh complete protease inhibitor cocktail tablets (Roche, Mannheim, Germany) and phosphatase (1 mM NaF, 1 mM Na_3_VO_4_) inhibitors. Lysates were boiled for 5 min, then sonicated at 100 Watt for 20 sec, and cell debris were removed by centrifugation 5 min at 12000×g. Total proteins were quantified according to Lowry et al. [23]. Equal amounts of proteins were fractionated on sodium dodecyl sulfate polyacrylamide gel electrophoresis (SDS-PAGE) according to Laemmli [24], and immunoblotted according to Towbin et al. [25] with the following primary antibodies: anti-YY1 (1∶1000; Santa Cruz sc-281), monoclonal anti-α-tubulin (1∶1000; Sigma, clone B-5-1-2, T6074) and monoclonal anti-β-actin (1∶1000; Sigma AC-15). After TBS-T washing, membranes were incubated with horseradish peroxidase-conjugated secondary antibody (BioRad, Hercules, CA) and peroxidase activity was detected by ECL (ECL Plus Kit, Amersham Biosciences, Arlington Heights, IL).

### Electrophoretic Mobility Shift Assay (EMSA)

Nuclear extracts were prepared by low salt/detergent cell lysis followed by high salt extraction of nuclei as reported [26]. Double-stranded oligonucleotides were 5′ end-labeled with [γ-^32^P] ATP (Perkin Elmer Life Sciences) and T4 polynucleotide kinase (T4 PNK, Roche Diagnostics). For direct binding experiments, nuclear extracts (5 µg) were preincubated with 3 µg of double-stranded non-specific DNA competitor poly(dI-dC) (Amersham Pharmacia Biotech) for 10 min on ice in binding buffer (20 mM Hepes-KOH, pH 7.9, 0.1 M KCl, 5% (v/v) glycerol, 0.2 mM EGTA, 0.2 mM EDTA, 1 mM dithiothreitol). After this time, a ^32^P-end-labeled DNA probe was added to the mixtures at a final concentration of 4 nM and the incubation was continued for an additional 30 min. Reaction mixtures were then submitted to electrophoretic separation on 5% native polyacrylamide gels (29∶1 cross-linked) in Tris-glycine buffer (25 mM Tris base, 192 mM glycine). DNA/protein complexes were detected by exposing the dried gel in a Molecular Imager (Bio-Rad). For competition experiments, nuclear extracts were incubated with a 50-fold excess of double-stranded competitor ODN for 10 min before adding the ^32^P-labeled probe. For supershift experiments, nuclear extracts were incubated with 1 µg of anti-YY1 (Santa Cruz sc-281 X) or anti-Egr1 (Santa Cruz sc-189 X) antibody for 30 min at room temperature prior to the addition of the radiolabeled probe.

### ChIP Assay

ChIP assays were performed using the ChIP assay kit essentially as described by the manufacturer (Upstate Biotechnology). Briefly, HeLa cells at ∼80% confluency were cross-linked with 1% formaldehyde for 10 min at room temperature. Cross-linking was stopped with the addition of 125 mM glycine (for 5 min at room temperature) and cold PBS washes. Cross-linked cells were centrifuged at 600×g for 10 min at 4°C and subsequently resuspended, at a concentration of 2×10^7^ cells/ml, in sodium dodecyl sulfate lysis buffer (Upstate) with protease inhibitors. Cross-linked DNA was subjected to ten 15 s sonication pulses at 43 watts, in a volume of 300 µl, by using a Labsonic 1510 Sonicator (Braun, Melsungen, Germany), in order to shear chromatin to an average size of between 200 and 500 bp. For each immunoprecipitation, aliquots of 100 µl, containing ∼2×10^6^ cell equivalents of sheared chromatin, were diluted to a final volume of 1 ml with ChIP buffer and incubated overnight at 4°C with 4 µg of anti-YY1 (Santa Cruz sc-281 X) or anti-Egr1 (Santa Cruz sc-189 X) control antibody, or with no antibody added. Ten microliters of diluted chromatin were saved and stored for later PCR analysis as 1% of the input extract. NaCl was added to the ChIP samples for 4 h at 65°C to reverse the cross-links. The input genomic DNA and the immunoprecipitated DNA weres treated with RNase and proteinase K and then extracted using the spin columns provided by the kit, according to the manufacturer’s protocol. For Western-ChIP analysis, 1/10 (20 µl) of the ChIPed samples after de-crosslinking was separated onto a 8% (w/v) polyacrylamide gel in parallel with a whole HeLa cell extract, as a positive control, and probed with the anti-YY1 antibody. Each experiment was repeated in triplicate.

### Quantification of ChIP Assay

For quantitative analysis of ChIP products, RealTime PCR was carried out using 5-µl aliquots of purified input genomic DNA and immunoprecipitated DNA and the SYBR green RealTime master mix. For the PCR survey of YY1 occupancy, the following primer pairs were employed: intron probe II, forward and reverse, reported above [19], to detect the +137/+336 intron sequence bearing the YY1 binding site; intron probe V, forward 5′-AGGGTAGGCTCTCCTGAATCGAC-3′ and reverse 5′-TCACAAAACACACTCGCCAACC-3′, which amplify the downstream intron region (+608/+766) [19]; U1 forward 5′-TGTGTGGGGTTTCCGCCTCT-3′ and U2 reverse 5′-CGCGGGACAAGGACAATGAC-3′, for amplification of the upstream *UbC* promoter region (−781/−636). Annealing temperature was 60°C for intron probe II and intron probe V Fwd/Rev primers and 68°C for the U1/U2 primer pair. PCR signals from immunoprecipitation samples were referred to their respective input signal to account for differences in DNA quantities before immunoprecipitation and primer efficiency. RealTime PCR data were analyzed according to the 2^−ΔΔ*C*^_T_ method [21]. Each quantitative PCR point was performed in triplicate and the average ± SE was calculated from three different ChIP analyses.

### Plasmid-based ChIPs

For plasmid-based ChIPs, transfections were performed in HeLa as follows: 8×10^5^ cells were plated overnight in 60-mm tissue culture dishes and grown to a 70–80% confluency. 1 µg of plasmid, bearing either wild-type or mutant YY1 sites in the cloned *UbC* promoter/intron region, was transfected as described above. The empty vector pGL3basic was transfected in parallel for purpose of background subtraction. ChIPs were performed as described above: for these studies 2×10^6 ^cell equivalents of sheared chromatin were used for each immunoprecipitation reaction with YY1 specific antibody (5 µg) or with no antibody added. 5-µl aliquots of 1 to 10 dilutions of each sample were used in triplicate for qPCR analysis using a plasmid backbone specific primer and a primer complementary to a portion of the *UbC* promoter/intron sequence. Primers employed to survey intronic YY1-e binding site occupancy were: RVprimer3 forward 5′-CTAGCAAAATAGGCTGTCCC-3′ and intron probe II reverse (sequence shown above). Additionally, each ChIP sample was also subjected to PCR with the primers for the luciferase cDNA sequence reported above, which served both as internal control for transfection efficiency and specificity of immunoprecipitation (data not shown). Data analyses were performed as described for standard ChIP assay. Results are representative of at least three independent experiments, assayed in triplicate.

### RNA Immunoprecipitation (RIP)

RNA immunoprecipitation was performed essentially as described in [27]. Briefly, 1×10^7^ HeLa cells per IP were crosslinked with 1% formaldehyde in PBS and collected by scraping. Cells were incubated in swelling buffer (5 mM Hepes pH 8.0, 85 mM KCl, 0.5% Nonidet P-40) supplemented with protease inhibitor cocktail (Roche), on ice for 10 min; then recovered by centrifugation and resuspended in nuclei lysis solution (50 mM Tris–HCl, pH 8.1, 10 mM EDTA pH 8.0, 1% sodium dodecyl sulphate) containing 40 U/ml RNase Inhibitor (Invitrogen), and protease inhibitors and kept on ice for 10 min. The extract was diluted tenfold with FA lysis buffer (1 mM EDTA pH 8.0, 50 mM HEPES–KOH pH 7.5, 140 mM NaCl, 0.1% sodium deoxycholate, 1% triton X-100), supplemented with protease and RNase inhibitors and then sonicated at 43 watts for 5 min with 30 s on/off cycles, by using the Labsonic 1510 Sonicator, described above. After preclearing with Protein G agarose (1 h at 4°C) the supernatant was treated with 60 U/ml RNase-free DNAse I (Roche) for 20 min at 37°C. After centrifugation, the supernatant was incubated overnight at 4°C with 5 µg of anti-YY1 (Santa Cruz sc-281 X) or IgG antibodies. Ten microliters, i.e. 1% of the total sample volume corresponding to an extract equivalent to 1×10^5^ cells, were saved to prepare input RNA. Protein G agarose (50 µl of a 50% slurry) was added to the RIP samples and incubated with rotation for 90 min at 4°C. Beads were washed four times as detailed in the Protocol without modifications [27]. Elution was performed twice with 75 µl of RIP elution buffer (10 mM EDTA, 100 mM Tris–HCl pH 8.0, 1% sodium dodecyl sulfate, 40 U/ml RNase inhibitor).The pooled eluates were treated with 20 µg of proteinase K (for 1 h at 42°C and then 1 h at 65°C). RNA was recovered by phenol-chloroform extraction and treated with 2 U of TURBO DNase (30 min at 37°C). The total RNA recovered was concentrated and reverse transcribed with oligo-dT and random primers using SuperScript® First-Strand Synthesis System. Control reactions without reverse transcriptase were also prepared.

qPCR was carried out using 5-µl aliquots of a 1 to 7 dilution of cDNAs and the following primer pairs: *UbC*, forward and reverse and intron probe II, forward and reverse (reported above); exon 1, forward 5′-GGGATTTGGGTCGCGGTTC-3′ and reverse 5′-TGACGATCACAGCGATCCAC-3′ (3.5 mM MgCl_2_, 45 bp); 18S rRNA, forward 5′-GTAACCCGTTGAACCCCATT-3′ and reverse 5′-CCATCCAATCGGTAGTAGCG-3′ (2.5 mM MgCl_2_, 151 bp).

The immunoprecipitation efficiency for each specific fragment was calculated by dividing the amount of product obtained with the immunoprecipitated RNA by the amount obtained with the input RNA. The RIP enrichment (fold increase) was derived by dividing the IP efficiency of the YY1 RIP by the IP efficiency of the control IgG RIP.

### Computational Analysis and Statistics

For bioinformatic analysis of the *UbC* intron region, MatInspector [28] (http://www.genomatix.de/matinspector.html) and TESS [29] (http://www.cbil.upenn.edu/cgi-bin/tess/tess) softwares were used. Statistics were analyzed by one-way ANOVA with Tukey post-tests for multiple comparisons or by two-tailed Student’s t test for pairwise comparisons using GraphPad Software (La Jolla, CA).

## Results

### Identification of Sp1 and YY1 Binding Sites in the *UbC* Intron Sequence

We previously cloned the upstream sequence of the human ubiquitin *C* gene and found that the maximal promoter activity is achieved when to the proximal promoter (PP) region of 371 nt, is added the unique 5′-UTR intron, which is crucial for basal transcriptional activity (Figure 1) [19]. Inspection of the overall intron sequence by computer-based analyses with MatInspector and TESS softwares, revealed the presence of multiple Sp1 and YY1 binding motifs. Electrophoretic mobility shift assay revealed the presence of multiple *cis-*acting elements for Sp1 and YY1 transcription factors, within specific intron sequences, which we previously termed ODN 1 (nt +158/+212 respect to transcription start site), ODN 3 (nt +418/+467), ODN 5 (nt +645/+679), probe II (nt +137/+336) and probe IV (nt +445/+655) [19]. The exact positioning of Sp1 and YY1 binding sites within ODN 3 was determined in the previous paper [19].

**Figure 2:**
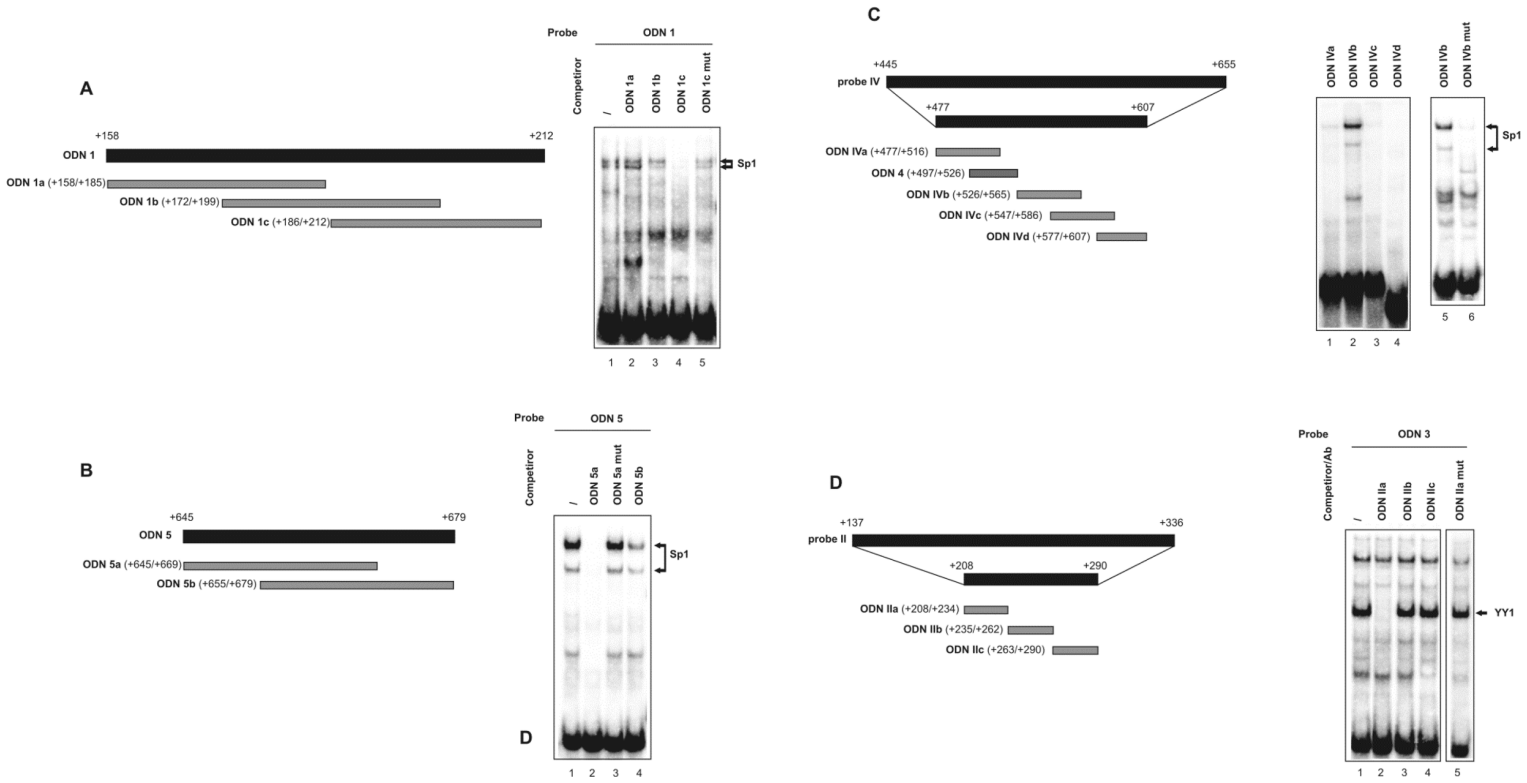
Identification of Sp1 and YY1 binding sites within the intron sequence, by EMSA. (**A**, *Left*) Schematic representation of full-length ODN 1 and of the three overlapping duplexes referred to as ODN 1a, ODN 1b and ODN 1c (28-, 28- and 27-bp long, respectively). Numbers in brackets refer to the position with respect to the major transcriptional start site, identified as +1. (*Right*) EMSA was performed with ^32^P-labeled ODN 1 as the probe. HeLa nuclear extract (5 µg) was preincubated in the absence (–) or presence of a fifty-fold excess of wild-type or mutant competitors, before addition of the labeled probe, as indicated. (**B**, *Left*) Full-length ODN 5 and the two overlapping duplexes, 25-bp long, referred to as ODN 5a and ODN 5b. (*Right*) EMSA was performed as reported in **A**, with ^32^P-labeled ODN 5 probe, in the absence (–) or presence of a fifty-fold excess of wild-type or mutant competitors, as indicated. (**C**, *Left*) Schematic representation of full-length and partial intron probe IV and of the four overlapping duplexes designed on the partial region and used for direct binding in gel shift experiments (ODN IVa, ODN IVb, ODN IVc and ODN IVd). ODN 4 used in previous studies was reported as well [19]. (*Right*) Direct binding of HeLa nuclear extract (5 µg) with the ^32^P-labeled ODNs (lanes 1–4) and demonstration of binding specificity to ODN IVb by using ODN IVb mut as the probe (lanes 5–6). (**D**, *Left*) Schematic representation of full-length and partial intron probe II and of the three overlapping duplexes designed on the partial region (ODN IIa, ODN IIb and ODN IIc), used in competitive EMSA experiments. (*Right*) EMSA was performed with ^32^P-labeled ODN 3 as the probe, in the absence (–) or presence of a fifty-fold excess of wild-type or mutant competitors, as indicated. Arrows point to the Sp1 (**A**, **B** and **C**) or YY1 (**D**) bound probe. Representative EMSA are shown. Experiments were repeated three times with similar results.

As previously determined, ODN 1, ODN 5 and probe IV were able to form complexes with Sp1 protein factor [19]. To identify nucleotides critical for Sp1 binding, the ODN 1 sequence was dissected into three double-stranded oligonucleotides (ODN 1a, ODN 1b and ODN 1c) respectively 28-, 28- and 27-bp long, with a 14-bp overlap, to be used as competitors versus full-length ODN 1 (Figure 2A). Competitive EMSA demonstrated that ODN 1c abrogated nucleoprotein binding to the ODN 1 probe, while its mutant version, bearing the GGG→ACA changes in the putative Sp1 binding motif, did not interfere (Figure 2A). No competition was observed with ODN 1a and ODN 1b (Figure 2A).

Similarly, ODN 5 was fragmented into two overlapping sequences 25 bp-long (ODN 5a and ODN 5b; Figure 2B), tested as competitors versus ODN 5. As shown in the representative EMSA, ODN 5a prevented protein/DNA complex formation, while the corresponding mutagenic sequence was ineffective, like ODN 5b (Figure 2B). This result confirms that GGGAGG is an Sp1 binding site, as indicated by computational analysis.

To identify the Sp1 binding site(s) within intron probe IV, we considered only the sequence corresponding to nt +477/+607, which is devoid of the outer parts previously investigated and excluded [19]. The remaining sequence was dissected into four overlapping fragments (ODN IVa, ODN IVb, ODN IVc and ODN IVd), depicted in Figure 2C, which were radiolabeled and incubated with HeLa nuclear extracts in direct gel shift experiments. Sequence referred to as ODN IVb generated retarded bands with a typical Sp1 pattern, and when nucleotide changes were introduced in the putative Sp1 binding motif (GGGTGG→ACATGG), the specific nucleoprotein complex disappeared (Figure 2C). No retarded bands were generated following incubation of HeLa nuclear factors with ODN IVa, ODN IVc and ODN IVd (Figure 2C). Thus, probe IV contains the unique Sp1 site, identified within ODN IVb.

We have previously published that the *UbC* intron, besides Sp1 proteins, also interacts with YY1 transcription factor at least with two binding sites, which fall within ODN 3 and probe II. YY1 binding motif within ODN 3 was previously characterized [19], the identification of the one(s) in probe II was addressed in the present work. Probe II intron sequence (nt +208/+290), deleted of the outer parts which failed to generate DNA/protein complexes [19], was dissected into three fragments (referred to as ODN IIa, ODN IIb and ODN IIc), designed for competitive gel shift experiments (Figure 2D); ODN 3 was selected as the probe. Results from EMSA assay demonstrated that a 50-fold molar excess of ODN IIa interferes with the formation of the prominent fastest YY1 complex, whereas the same excess of both ODN IIb and ODN IIc did not affect transcription factor binding (Figure 2D). Inspection of the competitive sequence element, indeed revealed a match to the consensus binding site for the ubiquitously expressed YY1 transcription factor (ATGGCGG) [30]. ODN IIa carrying mutations that destroy the core YY1-binding site (AGTGCAC), failed to disrupt the protein complex (Figure 2D), thus confirming the specificity of the interaction observed.

### Point Mutation Studies of Putative Transcription Factor Binding Sites: Significance of YY1 Binding Sites

To determine the significance of the identified Sp1- and YY1-binding sites, we introduced mutations in these sites individually or simultaneously into the P3 promoter-reporter construct. The P3 construct contains 371 nt upstream of the transcription start (herein referred to as proximal promoter, PP) and the 5′-UTR region, composed of the 63-nt exon 1 and the 812-nt unique intron of the *UbC* gene (Figure 1) [19]. P3 was chosen as the wild-type reporter construct for mutagenesis experiments because it displayed the highest promoter activity with the smallest upstream promoter sequence. To design mutagenic primers, we considered the nucleotide substitutions that made competitor ODNs unable to bind transcription factors in EMSA.

**Figure 3:**
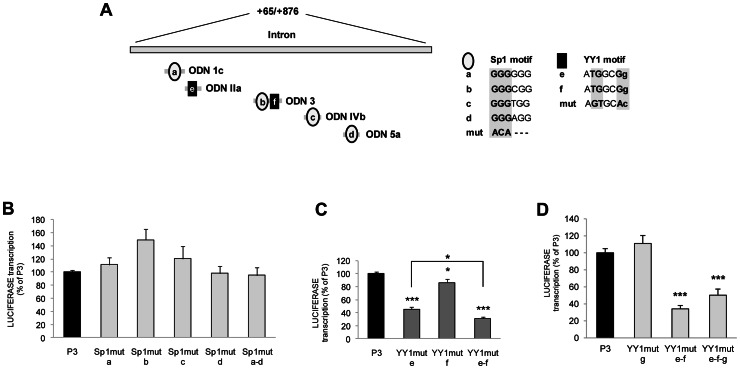
Mutagenesis of YY1, but not Sp1, intron binding sites negatively affects *UbC* promoter activity. (**A**) The schematic diagram shows the *UbC* intron region (nt +65/+876). ODNs and the relative positions of the putative transcription factor binding motifs are illustrated below: Sp1 binding sites are represented by open ovals and identified with a single-letter code, from (a) to (d); YY1 binding sites are represented by filled rectangles and named (e) and (f). Sequences of the different Sp1 and YY1 binding sites are shown and nucleotide substitutions introduced by mutagenesis are highlighted. (**B**) HeLa transient transfections with wild-type (P3) and the different Sp1 mutant luciferase constructs were carried out and luciferase expression evaluated by RT and quantitative RealTime-PCR at 48 h post-transfection as detailed under “Materials and Methods”. Promoter activity of the wild-type P3 was set to 100% and promoter activity of the mutants was expressed as a percentage of the wild-type construct. (**C**), (**D**) HeLa transient transfections with wild-type (P3) and the different YY1 mutant luciferase constructs were carried out and assayed as in **B**. Data presented are the means (±SE) of at least four different experiments, with two independent plasmid preparations. Asterisks indicate statistical differences (*, p<0.05; **, p<0.01; ***, p<0.001 versus P3). The statistically significant difference between YY1mut e–f and YY1mut e (**C**) is also indicated (*, p<0.05).

Regarding Sp1, we overall identified four binding sites (namely Sp1-a, -b, -c, -d) located, respectively, in ODN 1c, ODN 3, ODN IVb and ODN 5a (Figure 3A). In all sites, nucleotide substitutions were introduced in the first three bases of the Sp1 consensus motif (GGG to ACA). In agreement with the above results, no retarded bands were generated following incubation of HeLa nuclear extracts with probes obtained by amplifying the intron region with primer pairs encompassing the mutagenized Sp1 sites (data not shown). However, when single-site Sp1 mutant constructs were used to transfect HeLa cells, no decrease in luciferase expression (respect to the wild-type P3 construct) was detected, independently of the mutagenized site (Figure 3B). A reporter vector with mutations in all intron Sp1 binding motifs (referred to as Sp1mut a–d) was generated to test if the multiple redundant Sp1 binding sites could alternatively transactivate the *UbC* promoter, thus accounting for the lack of effect when only one site was inactivated. Upon transfection in HeLa cells, the whole mutagenized sequence exhibited the same promoter activity of P3 and of single-site Sp1 mutant constructs (Figure 3B). As a whole, these data suggest that Sp1 binding sites detected within the intron sequence are not responsible, *in vivo*, for the intron-mediated boost of *UbC* gene transcription.

Therefore, we focused our attention towards the potential role of the other *trans*-acting factor able to interact, *in vitro*, with the intron sequence: the Yin Yang 1 (YY1) transcription factor. Dissecting the *UbC* intron sequence by EMSA we found two identical YY1 binding sites (ATGGCGg), located within ODN IIa and ODN 3, in the antisense strand, and referred to as YY1-e and YY1-f, respectively. To examine the significance of the two YY1-binding sites for the expression of *UbC* gene, we generated mutations in one or both YY1 sites in the P3 promoter construct (Figure 3A) and confirmed by EMSA that mutations introduced effectively disrupted YY1 binding (data not shown). The analysis of reporter expression in HeLa cells revealed that the YY1-e mutation reduced the P3 promoter activity by 55±3%, while mutation of YY1-f site caused a smaller change in promoter activity, (around 15±6%) (Figure 3C). Simultaneous mutation of both sites reduced the activity by ∼70%, exhibiting an additive effect of the two mutations and suggesting that both sites are required for maximal activity (Figure 3C). In support to this conclusion, luciferase expression driven by YY1mut e and YY1mut e–f constructs showed a statistically significant difference (p<0.05).

In silico analysis of the proximal promoter and first exon sequence using TESS software revealed one putative YY1 binding site in the sense strand, at nt −165/−157 (GGACATtTT). The *in vitro* association of YY1 factor with the upstream target sequence was confirmed by EMSA and supershift assay (data not shown). To assess the functional role of this YY1 binding site (referred to as YY1-g) *in vivo*, point mutations were introduced in the YY1-g motif, in the P3 reporter vector. When the P3 carrying the single-site YY1 mutation (YY1mut g) was transiently transfected in HeLa cells, no decrease in luciferase expression (respect to the wild-type P3 ) was detected (Figure 3D), indicating that the upstream YY1 binding site does not participate to the promoter activity *in vivo*. In support to this conclusion, mutagenesis of YY1-g site in the YY1mut e–f construct did not further lower luciferase expression (Figure 3D).

### YY1 Binds to the Intron Target Sequences both *in vitro* and *in vivo*

**Figure 4:**
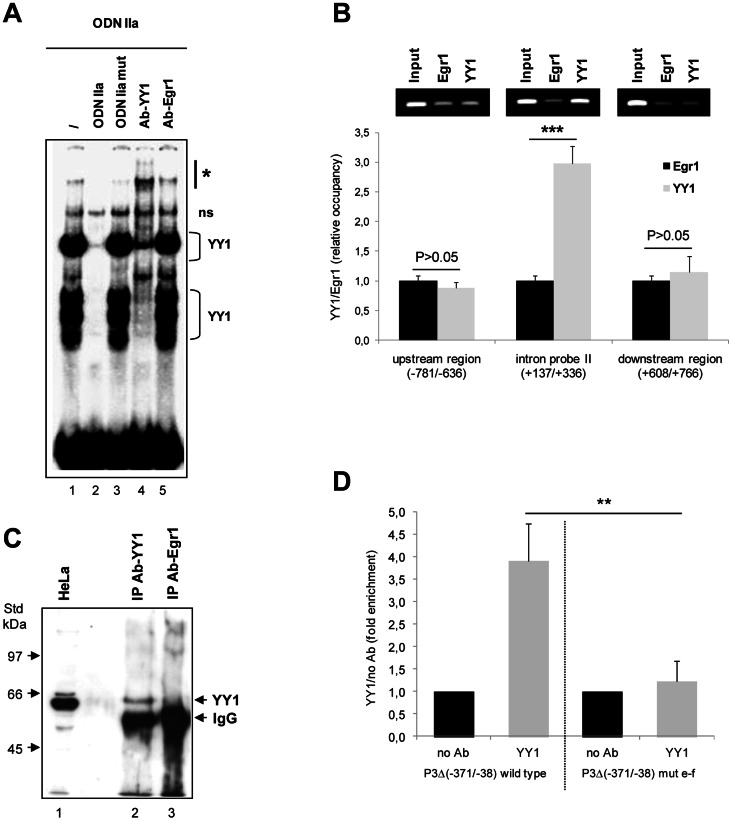
YY1 binds to the ATGGCGG intron sequences both *in vitro* and *in vivo*. (**A**) EMSA was performed with the ^32^P-labeled ODN IIa as the probe, in the absence (–) or presence of a fifty-fold excess of wild-type or mutant competitors, as indicated. To confirm binding specificity, 1 µg of YY1 or Egr1 antibody was preincubated with nuclear extracts, before addition of the radiolabeled probe. Arrow points to the YY1 bound probe and the asterisk indicates the supershifted complex; ns marks a non specific nucleoprotein complex. (**B**) ChIP analysis of association of YY1 nuclear protein with the *UbC* intron region was performed using chromatin from formaldehyde-crosslinked HeLa cells immunoprecipitated with YY1 and Egr1 specific antibodies. ChIPed DNAs were analyzed by quantitative RealTime PCR with primers detecting the intron probe II region (+137/+336) containing the most proximal YY1 site, the downstream intron region (+608/+766) and the upstream promoter region (−781/−636). Data in the graph represent the means (±SE) of three independent immunoprecipitations, analyzed in triplicate. Asterisks indicate the statistical difference, calculated using t-test, between YY1 and Egr1 ChIPed samples (***, p<0.001). Representative gel for each primer set is shown above the histogram. (**C**) For Western-ChIP analysis, 20 µl of ChIP samples after de-crosslinking (lanes 2 and 3) were separated onto a 8% (w/v) polyacrylamide gel in parallel with a whole HeLa cell extract (lane 1) as a positive control, and probed with the anti-YY1 antibody. The gel photograph is representative of three repeated experiments, with similar outputs. The arrows on the right point to the YY1 and IgG immunoreactive bands. Molecular weight standards are indicated on the left. (**D**) Plasmid ChIPs were performed on HeLa cells transfected with P3Δ(−371/−38) plasmid, bearing either wild-type or mutant YY1 sites in the intron region. After 48 h ChIPs were performed with 5 µg of YY1 specific antibody or with no antibody added as a negative control to provide baseline values. After ChIP, the −37/+336 region of the human *UbC* promoter was amplified using RealTime qPCR with a plasmid backbone specific primer (RVprimer3 forward 5′-CTAGCAAAATAGGCTGTCCC-3′) and a primer complementary to a portion of the *UbC* promoter/intron sequence (intron probe II reverse 5′-CGCATTAGCGAAGGCCTCAAG-3′). PCR signals, representing YY1 occupancy, are normalized to the input and plotted as fold enrichment over the no Ab control. Results shown are the means (±SE) of three separate experiments, assayed in triplicate. The asterisks represent significant difference of wild-type versus mutant P3Δ construct (**, p<0.01).

**Figure 5:**
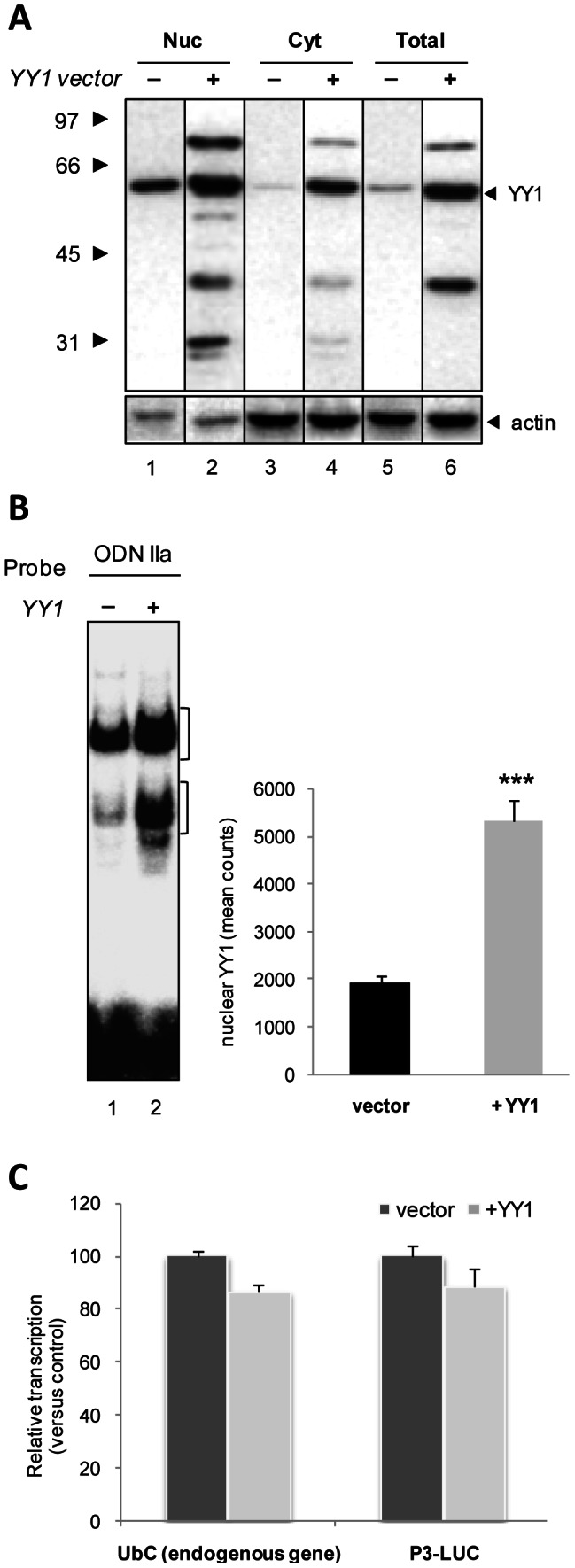
Effects of ectopic expression of YY1 on both reporter and endogenous target gene expression. (**A**) Immunoblotting of proteins from HeLa cells transfected with YY1 expression vector (lanes 2, 4, 6) or control empty vector (lanes 1, 3, 5), at 48 h post-transfection. Nuclear (Nuc, 10 µg), cytosol (Cyt, 20 µg) and total (20 µg) extracts were obtained as reported under “Materials and Methods”. Arrows mark the YY1 and actin bands (upper and lower panel, respectively). Molecular weight standards (kDa) are indicated on the left. Actin was employed as the endogenous internal control. A representative blot is shown. Experiments were repeated three times with similar results. (**B**) EMSA performed with ^32^P-labeled ODN IIa, containing a YY1 binding sequence, as the probe and HeLa nuclear extracts of cells transfected with control (-, lane 1) or YY1 expression vector (+, lane 2). The parentheses indicate the major nucleoprotein complexes. A representative image of three different EMSA is shown. Quantification of DNA-protein complexes was performed in a Molecular Imager and results are reported in the histogram as mean counts (±SE) of three different experiments (***, p<0.001). (**C**) Quantitative RealTime reverse transcription PCR analysis of endogenous ubiquitin *C* and luciferase mRNA levels in cells receiving YY1 expression plasmid (+YY1) or control empty vector (vector), performed at 48 h post-transfection. All the values are the means (±SE) of five different experiments.

In the previous work, we obtained the direct evidence for the binding, *in vitro*, of YY1 protein to ODN 3 intron sequence (herein referred to as YY1-f site) [19]. In this study we sought out to determine if the protein factor binding the putative YY1-e site is indeed YY1 transcription factor: EMSA was then performed using radiolabed ODN IIa as the probe. A prominent band and some faster migrating complexes were formed upon incubation with HeLa nuclear extract (Figure 4A). These protein/DNA complexes were successfully competed away by a 50-fold molar excess of ODN IIa cold probe, but not by a mutant competitor, carrying specific nucleotide changes within the YY1 target motif, confirming the specific binding of the YY1 protein to the probe. Moreover, the same complexes that were specifically competed away, disappeared and were partially supershifted by preincubating the nuclear extract with a specific YY1 antibody (Figure 4A). Conversely, the faint slower migrating band seems to be nonspecific, since it was not affected by addition of a specific ODN competitor and by YY1 antibody. No supershifted band was observed after the addition of anti-Egr1 antibody (Figure 4A). The gel has been overexposed to show the supershifted band, thus the intensity of the protein/DNA complexes does not reflect their relative abundance, which can be indeed appreciated in Figure 5B. Collectively, these results indicate that YY1 is the *trans-*acting factor binding to the ODN IIa probe, *in vitro*.

We next performed ChIP assay to verify whether YY1 nuclear protein binds to the endogenous ubiquitin *C* intron in intact HeLa cells. Chromatin immunoprecipitation analyses were carried out using a specific YY1 antibody to pull down cross-linked YY1/DNA complexes from HeLa cells. As an immunoprecipitation control, an unrelated antiserum, of the same isotype, recognizing Egr1 transcription factor, was used. Occupation of YY1 over the *UbC* intron region was detected by quantitative RealTime PCR performed on recovered DNA, using primers amplifying an intron fragment 200 bp-long (referred to as intron probe II) harboring the previously characterized proximal YY1 binding site (YY1-e). PCR was also performed using two further primer sets that detect, respectively, the downstream intron region (nt +608/+766) and the upstream promoter sequence (nt −781/−636). Because these sequences, lacking YY1 binding sites, should not be bound by YY1 factor, they were used as negative controls for the assay. Immunoprecipitation data are presented as the content of the different amplified fragments, on DNA purified from ChIPed samples with either YY1 or Egr1 antisera. As shown in the graph of Figure 4B, we found a 2.97-fold enrichment of the intron probe II sequence in the YY1-immunoprecipitated DNA sample, over its Egr1 isotype control (p<0.001), while no difference in the amplification signals was detected for both the upstream and the downstream region. Similar fold-change values for YY1 ChIP experiments were detected by others [31], [32]. Moreover, the results of ChIP performed with anti-Egr1 are similar to those obtained for the negative control where no antibody or IgG were added (not shown). The inset above the graph shows representative gels for each primer set used to amplify the immunoprecipitated and the input DNA samples.

Western blot analysis performed on samples from ChIP experiments, after the reverse cross-linking step, further demonstrated the specificity of the IP obtained with the anti-YY1 antibody (Figure 4C). Mutagenesis revealed that nucleotide substitutions in the intron YY1-binding site(s), mainly in the proximal one located within ODN IIa, interfere significantly with promoter expression (Figure 3C). DNA binding studies showed that YY1 protein factor binds to these sequences *in vitro* and strongly support that it may do so also *in vivo* (Figure 4A and B, respectively). On the whole these correlative data suggest that YY1 may have a functional role in the intron-dependent enhancement of *UbC* expression.

The requirement of ATGGCGG motifs for YY1 binding to the *UbC* intron region was directly addressed by plasmid ChIP assay in a cell culture model. Earlier groups have successfully established transcription factor binding to transiently transfected plasmid DNA (which becomes partially chromatinized) using plasmid ChIP [33]. The construct devoid of almost all the proximal promoter region, but retaining the entire intron spanning sequence was generated as described in ”Materials and Methods” and referred to as P3Δ (−371/−38). Plasmids P3Δ containing either the wild-type YY1 intronic binding sites or a mutant version in which the two ATGGCGG motifs have been mutagenized to AGTGCAC were separately transfected into HeLa cells. The promoter-less pGL3 reporter vector, transfected in parallel, provided the background values. After 48 h, cells were harvested and ChIP was performed using YY1-specific antibody, while the no-antibody sample served as a negative control. After ChIP, the region corresponding to the −37/+336 portion of the human *UbC* promoter/intron sequence, contained in the transfected plasmids, was amplified using RealTime quantitative PCR. The primer pair selected was such that only the *UbC* sequence present in the plasmid would be amplified but not the endogenous gene of HeLa cells used for transfections. PCR signals from YY1 and no antibody ChIPed samples were normalized to their respective input signals and then plotted as fold enrichment of YY1 IP over the no antibody control. The values obtained for pGL3 backbone were subtracted as the background. Fig. 4D shows a 3.90 (±0.84 SE)-fold enrichment of YY1 occupancy of the wild-type *UbC* intron region, whereas only a 1.22 (±0.46 SE)-fold enrichment was observed for the construct carrying the AGTGCAC mutated sequences. Thus, the above data indicate that YY1 in fact binds to the *UbC* intron region and that the ATGGCGG motifs are required for binding.

### Effects of YY1 Overexpression and Silencing on both Reporter and Endogenous Gene Expression

To more directly test for the importance of YY1 in both luciferase and *UbC* transcription, and above all in the intron-mediated enhancing effect, we carried out two functional studies: the ectopic expression of YY1 nuclear factor and the knockdown of endogenous YY1. The effects on both reporter and endogenous target gene expression were evaluated.

In the first assay, we caused the overexpression of YY1 in HeLa cells, by transfection of an expression vector carrying its cDNA under the control of a CMV promoter, while an empty vector was used as a control.

As expected, transfection of YY1 cDNA resulted in a marked increase (2.5-fold induction) of the 60 kDa migrating YY1 protein band (Figure 5A). Accordingly, the higher level of YY1 expression resulted in a ∼2.7-fold increase of YY1 binding to the YY1 consensus motif *in vitro*, as assessed by gel shift (Figure 5B). Upon YY1 overexpression additional immunoreactive bands were detected both in the lower and the higher molecular weight range (Figure 5A). While the faster migrating bands could be degradation products, we hypothesized that the higher molecular weight band could represent post-translationally modified YY1. Significantly, the presence of the higher YY1 form did not affect the DNA binding ability of the 60 kDa unmodified YY1 (which is the predominant form upon overexpression), nor seemed to bind DNA, as revealed by EMSA assay; indeed no additional bands were detected in YY1 overexpressing cells compared to untransfected cells. For all these reasons and because the study of YY1 post-translational modifications was beyond the scope of the present paper, this aspect was not further investigated.

Unexpectedly, both P3-directed luciferase and endogenous *UbC* expression were not affected by ectopic expression of YY1 transcription factor in HeLa cells (Figure 5C). By contrast, transient transfection of the same construct negatively regulated dystrophin transcription in C2C12 muscle cells [20], thus demonstrating that construct-driven overexpression of YY1 leads to a transcriptional competent protein factor. Therefore, it could be speculated that the lack of induction, under our experimental conditions, could be due to the fact that YY1 is already highly expressed in this cell line [34], [35] and its binding sites within the *UbC* promoter are probably almost fully occupied *in vivo*.

**Figure 6:**
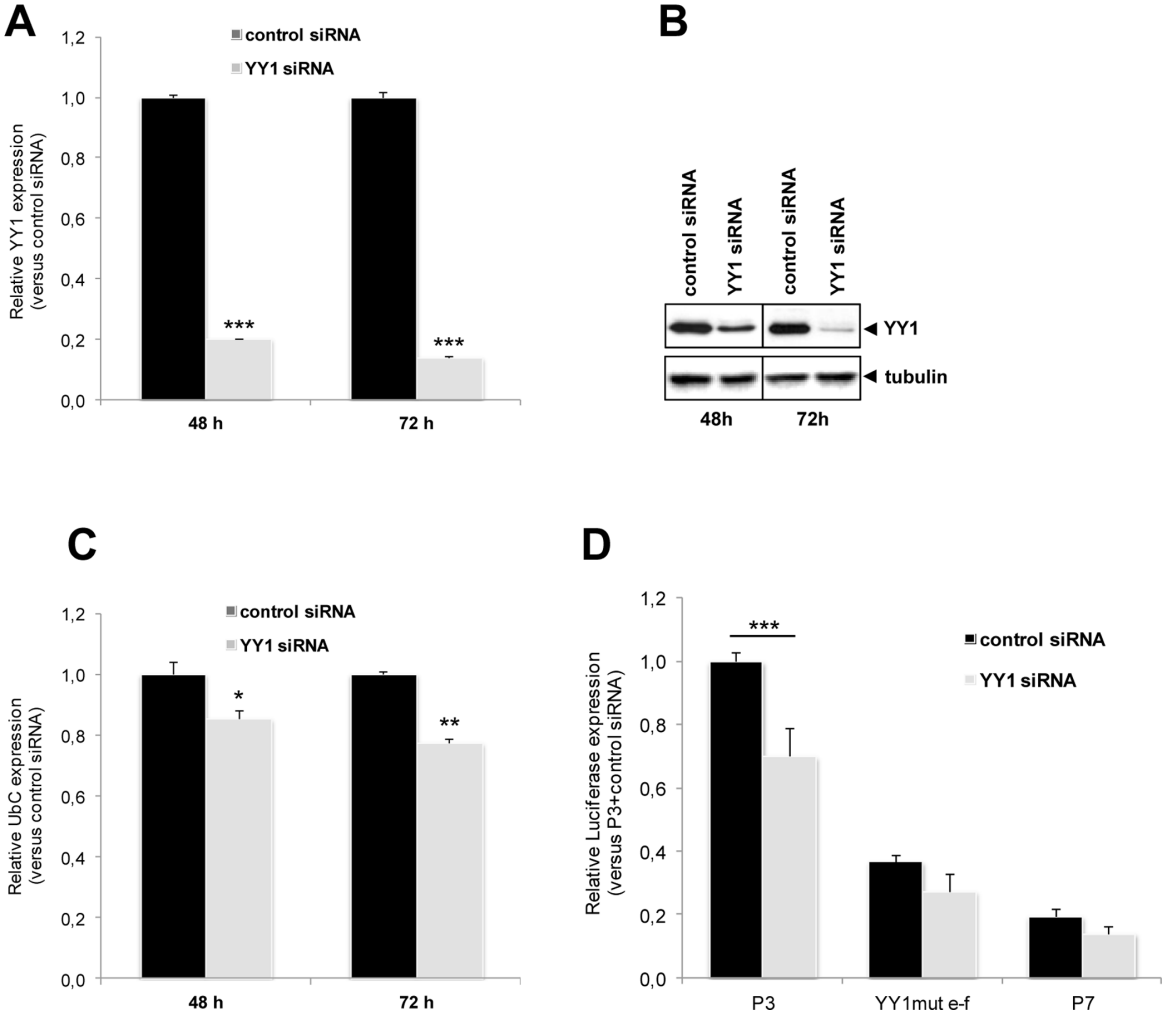
Effects of YY1 knockdown on both reporter and endogenous target gene expression. (**A**) Efficiency of siRNA-mediated knockdown of YY1. HeLa cells were transfected with control nonsilencing siRNA oligo or with a YY1-specific siRNA. Quantitative RealTime reverse transcription PCR assays were done 48 and 72 h post-transfection. Expression data, normalized to B2M, were analyzed by the 2^−ΔΔ*C*^_T_ method and referred to the control, set equal to 1. Results shown in the graph are the means (±SE) of ten independent experiments. Asterisks indicate statistical significance versus control siRNA transfected cells, at each time point (***, p<0.001). (**B**) Western immunoblot of proteins from HeLa cells transfected with control or YY1-specific siRNA, at 48 and 72 h post-transfection. Equal amounts of total cellular proteins (20 µg) were loaded and immunoblotted for YY1. Blot was reprobed with α-tubulin as a loading control. The image is representative of three different experiments, with similar results. (**C**) Effects of YY1 depletion on expression of endogenous *UbC* gene. HeLa cells transfected with control or YY1-specific siRNA were analyzed by quantitative reverse transcription PCR, at 48 and 72 h post-transfection, for ubiquitin *C* RNA level. Expression data, normalized to B2M, are relative to the value of the control siRNA sample, set equal to 1. The histogram shows the means (±SE) of five different experiments. As in (**A**), asterisks indicate statistical significance versus control siRNA transfected cells, at each time point (*, p<0.05; **, p<0.01). (**D**) Effects of YY1 depletion on the *UbC* promoter-directed luciferase expression in HeLa cells. Cells cotransfected with P3, YY1mut e–f, or P7 reporter construct and control or YY1-specific siRNA, as labeled, were harvested at 72 h post-siRNA delivery and luciferase RNA level measured by Quantitative RealTime reverse transcription PCR. Expression data are relative to the value of P3 reporter vector in the control siRNA sample, set equal to 1. The graph shows the means (±SE) of at least ten independent experiments. Asterisks indicate statistical significance (***, p<0.001).

If YY1 is actually a positive regulator of *UbC* expression, decreasing YY1 levels should repress it. To test this hypothesis, we transfected HeLa cells with siRNAs against YY1 or, as a control, with a fluorescent labeled aspecific siRNA (AF 488). Two YY1-specific siRNAs (YY1_1 and YY1_3) were used to account for possible off-target effects and for statistical purposes as well. YY1 mRNA and protein levels were quantified 48, 72 and 96 h after transfection. Real-Time PCR assay revealed that YY1 mRNA was reduced by 80% at 48 h and the knockdown was even higher at 72 and 96 h (∼86%) (Figure 6A). Both specific siRNAs were similarly effective in reducing the YY1 mRNA level at every time point, whereas the control siRNA did not affect YY1, even at the long-lasting treatment. Western analysis showed a parallel significant reduction of YY1 protein expression, with the most prominent effect observed at 72 h post-transfection (Figure 6B). Knockdown of YY1 caused *UbC* expression levels to fall by approximately 15 and 23% at 48 and 72 h, respectively (Figure 6C).

To demonstrate that downregulation of *UbC* expression upon YY1 silencing is correlated to the identified intronic YY1 binding sites, we measured P3-directed luciferase expression upon knockdown of endogenous YY1 protein. The histogram (Figure 6D) shows a 30% statistically significant decrease of luciferase mRNA in cells treated with YY1-specific siRNA respect to cells receiving nonsilencing control siRNA (p<0.001; n = 10). By contrast, no statistically significant differences were observed for the construct carrying mutagenized YY1 binding sites (YY1mut e–f) and for the construct lacking the intron sequence (P7), cotransfected as controls (p>0.05; n = 10; Figure 6D).

### YY1 Intron Binding Sites do not behave as a Typical Enhancer Element

To shed more light on the mechanism of YY1-mediated activation of *UbC* promoter, we investigated whether it was able to positively affect transcription in a position- and orientation-independent fashion, which are typical features of enhancers [13].

**Figure 7:**
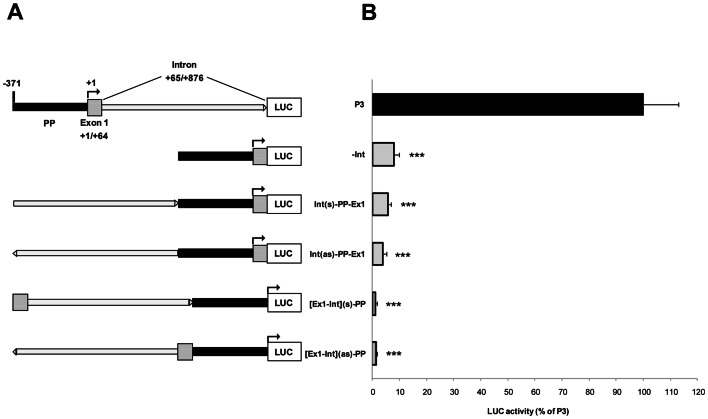
*UbC* intron does not act as a typical transcriptional enhancer. (**A**) Schematic representation of the tested DNA constructs. *UbC* promoter elements contained in the reference construct P3 are the proximal promoter (PP, black solid line, nt −371/−1), the first noncoding exon 1 (Ex1, hatched box, nt +1/+64), and the 5′-UTR intron (Int, open line, nt +65/+876). –Int, construct devoid of the intron sequence. Int(s)-PP-Ex1 and Int(as)-PP-Ex1, constructs where the *UbC* intron sequence was moved upstream of the proximal promoter, either in the sense or antisense orientation. [Ex1-Int](s)-PP and [Ex1-Int](as)-PP, constructs where the exon 1-intron cassette was moved upstream of PP, in both sense and antisense orientation. (**B**) Constructs displayed in **A** were transiently transfected in HeLa cells and luciferase activities were measured 48 h afterwards, normalized on total protein content, and referred to the P3 construct, set equal to 100%. The graph data are the means (±SE) of five independent experiments. Statistical analysis revealed a highly significant difference (***, p<0.001) between P3 and all other constructs investigated.

The *UbC* intron was placed upstream of the proximal promoter in the construct P3, in both sense and antisense orientation, generating the constructs referred to as Int(s)-PP-Ex1 and Int(as)-PP-Ex1, respectively (Figure 7A). Reporter vectors where the exon 1-intron cassette was moved upstream of the P3 promoter sequence, in both orientations ([Ex1-Int](s)-PP and [Ex1-Int](as)-PP, Figure 7A) were prepared to take into account the possible interactions between intron-bound YY1 and nuclear factor(s) binding to the exon 1. The –Int (minus intron) construct lacks the intron and corresponds to the previously described P7 plasmid [19]. All the reporter vectors shown in Figure 7A were transiently transfected in HeLa cells, and luciferase activity determined at 48 h, and referred to the one measured for the reference P3, set at 100%.

Results obtained showed that, compared to P3, all other constructs exhibited much lower luciferase expression (Figure 7B). Luciferase activity measured when the intron sequence was placed upstream of the promoter region, in both orientations, was similar to that of the construct devoid of the intron sequence (around 8% of P3). Constructs where both intron and exon 1 were moved upstream of the transcription start site, showed an even greater drop of luciferase activity (from 70- to 90-fold lower respect to P3 value). Similar results were obtained when the luciferase expression was evaluated at transcriptional level by RealTime PCR assay on total cellular RNAs reverse transcribed with random hexamers. These data strongly indicate that the stimulatory effect of the intron is not maintained when it is moved upstream of the promoter, outside the transcribed sequence, in either sense or antisense orientation. Thus the 5′-UTR intron is crucial for a sustained gene expression, only if maintained in its natural location.

Moreover, although almost no expression was detected when the intron was removed, or simply moved to a different position, the intron was unable to support expression by itself, in the absence of the enhancer elements of the proximal promoter, as found upon 5′-deletion of sequence −371/−38 in the “full-length” P3 to obtain the P3Δ construct (unpublished results).

On the whole, these data suggest that the *UbC* intron does not act as a typical transcriptional enhancer, given its inability to demonstrate any activity when moved upstream of the promoter region, and rather support the hypothesis that the intron acts by a process termed intron-mediated enhancement (IME).

### Splicing is Essential for *UbC* Intron-mediated Enhancement

**Figure 8:**
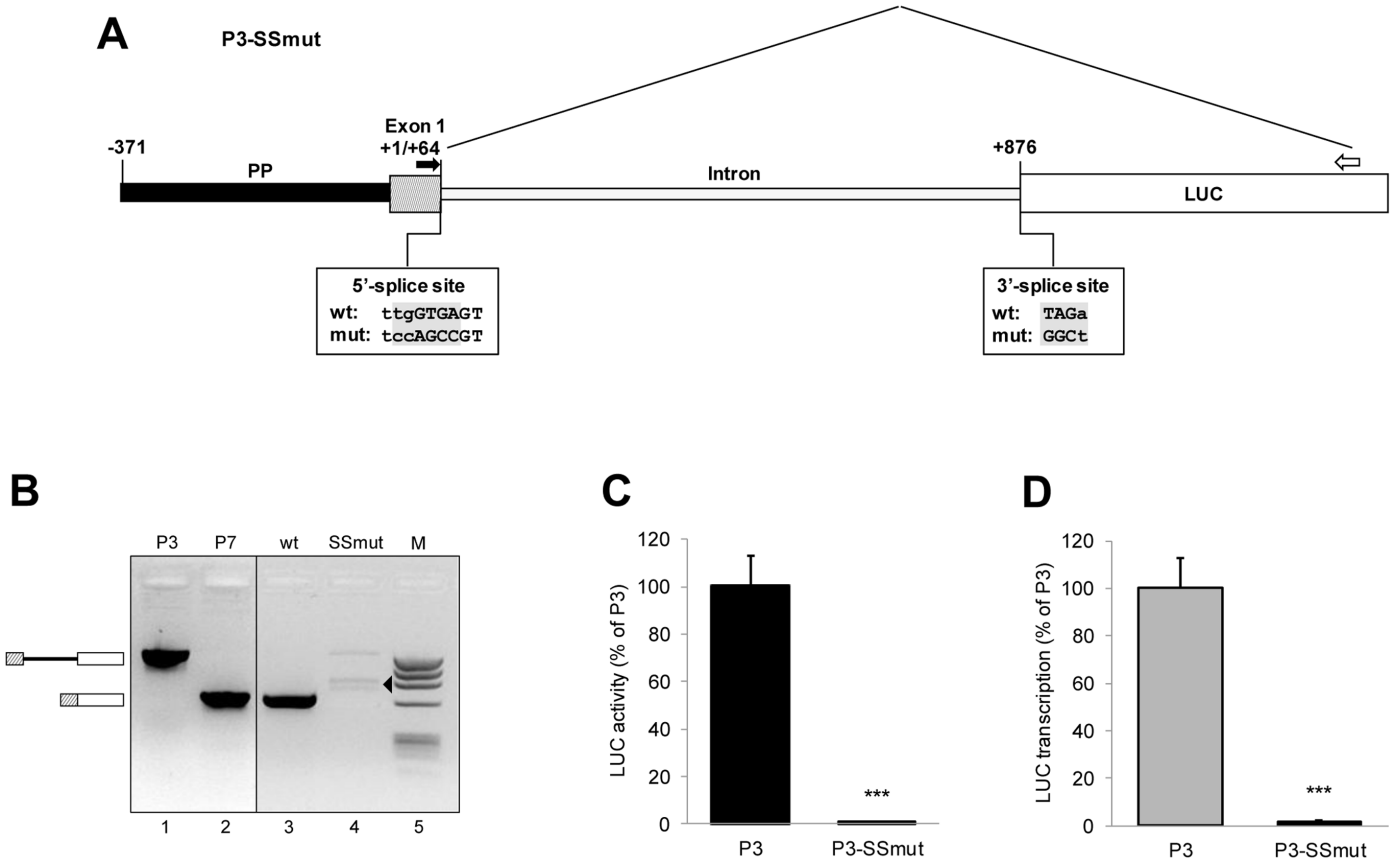
Effect of splice site mutations on P3-directed luciferase expression. (**A**) Schematic representation of the tested P3-SSmut construct. P3-SSmut is identical to the reference construct P3, except that the sequences of the 5′ and 3′ splice sites are mutated. The wild-type and mutant splice sites are displayed below the intron boundaries and the introduced nucleotide substitutions are highlighted in gray. The primers, which bridge the intron sequence, used for assessment of splicing by RT-PCR are indicated on the illustration: the forward primer was derived from the first exon of the 5′-UTR of *UbC* gene (black filled arrow) and the reverse primer is complementary to the luciferase (LUC) coding region (open arrow). (**B**) Gel image showing the results of RT-PCR analysis. The templates were cDNAs derived from P3 (wt, lane 3) or P3-SSmut (SSmut, lane 4) transfected cells, except for samples loaded in lane 1 and lane 2, where plasmids P3 and P7 were amplified in parallel to provide the size of the PCR products expected from unspliced (1416 bp) or spliced (651 bp) transcripts, respectively. M, DNA molecular weight marker (lane 5). The boxes separated by a line (on the left) indicate the expected size of unspliced transcripts, while the two adjacent boxes indicate the size of correctly spliced transcripts. The arrowhead highlights aberrantly spliced non specific products. The analysis is qualitative, but not quantitative, that is band intensity does not accurately reflect the relative abundance of the different transcripts. (**C**) Relative luciferase activity and (**D**) quantitative RealTime RT-PCR analysis of luciferase mRNA level from HeLa cells transfected with P3-SSmut or the wild-type construct P3. Data shown in the histograms are the means (±SE) of five independent experiments. Statistical analysis (t-test) revealed a highly significant difference (***, p<0.001) between P3- and P3-SSmut-directed luciferase expression.

Intron-mediated enhancement may take place at both transcriptional and post-transcriptional step, and in this last phase most of the IME effects are often linked to mRNA splicing [36]. Thus, to gain insights into the *UbC* intron features that sustain the enhancement of gene expression, we first investigated the effect of mutations in the splice recognition sites, in our transient reporter expression system. The construct named P3-SS (Splice Site)mut was created by site-directed mutagenesis of both 5′- and 3′-splice consensus motifs (Figure 8A). The sequence of the 5′-splice site 5′-tgGTGA-3′ was mutated to 5′-ccAGCC-3′, while the 3′-splice site 5′-TAGa-3′ was changed to 5′-GGCt-3′. Effective splicing inhibition upon splice site mutations was actually verified in HeLa cells independently transfected with P3 and P3-SSmut plasmids, by RT-PCR amplification with primers flanking the intron, as indicated in Figure 8A. Plasmids P3 and P7 were amplified in parallel as controls for the unspliced and spliced products, respectively (Figure 8B). Cells expressing the wild-type construct P3 showed a product with the expected spliced size (651 bp, Figure 8B), indicating that splicing occurs correctly. Cells expressing the construct in which splice sites were eliminated, showed a product with the expected unspliced size (1463 bp, Figure 8B), demonstrating that splicing is indeed impaired by the introduced mutations. Somewhat smaller nonspecific bands were also detected (Figure 8B, arrowhead) and they could reflect the usage of cryptic splice sites, as suggested by sequence analysis. The absence of PCR products in the negative RT control confirmed that the PCR fragments were entirely derived from cDNA and rules out any possibility that the amplified bands were the result of an artifact due to plasmid contamination (not shown).

Eliminating the splicing ability of the *UbC* intron in the P3-SSmut construct resulted in a drastic drop of luciferase activity, to a level below 1% of that of P3 (Figure 8C). To confirm that the low level of luciferase activity, observed upon transfection of splicing defective plasmid, was related to a reduction of the transcript amount, we performed a quantitative RealTime RT-PCR analysis, which revealed a significantly reduced steady-state luciferase mRNA level in P3-SSmut transfected cells (Figure 8D). Overall, these data indicate that impairing splicing significantly alters the *UbC* intron-mediated enhancing effect, i.e. intron ability to increase reporter gene expression.

### YY1 Intron Binding Sequences and Protein Factor are Required for Full Splicing Efficiency

The results so far described indicate that the ability of the *UbC* intron to enhance reporter gene expression is dependent upon both its position with respect to the transgene and the possibility to be spliced out correctly. Mutagenesis clearly uncovered that wild-type YY1 binding motifs are essential to sustain the intron-mediated increase of luciferase mRNA transcripts.

**Figure 9:**
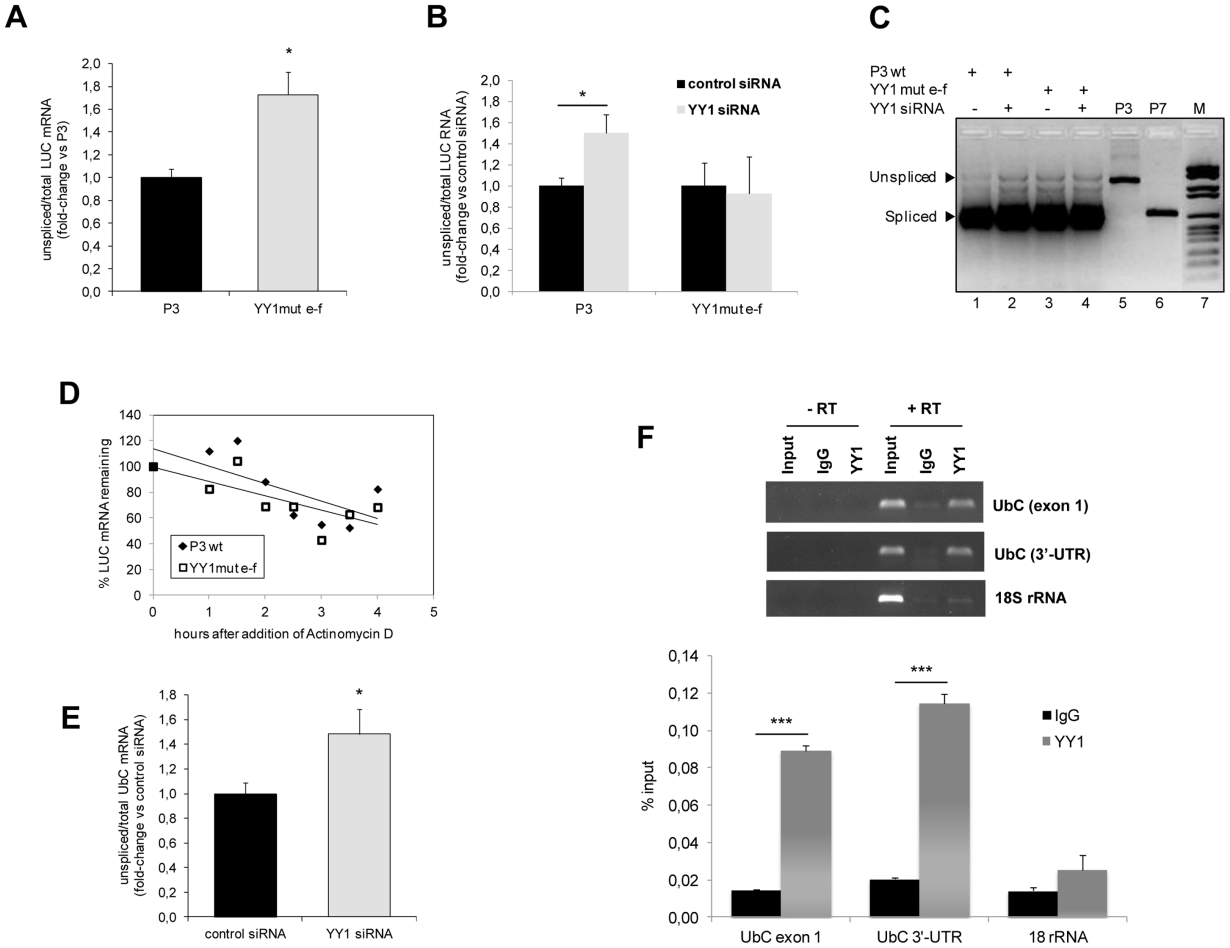
Role of YY1 binding sequences and *trans*-acting factor in the splicing of the *UbC* intron. (**A**) Absolute quantitative RealTime reverse transcription PCR for detection of unspliced luciferase transcripts upon mutagenesis of YY1 intronic binding sequences. HeLa cells transfected with the construct YY1mut e–f (carrying mutations in both YY1 binding sites) and with the wild-type P3 were harvested 48 h post-transfection and subjected to total RNA extraction, cDNA synthesis with random hexamers, and absolute quantification assay with two different primer pairs: LUC Fwd and LUC Rev, complementary to internal sites of luciferase coding sequence, were used to quantify total luciferase RNA copies (spliced and unspliced); intron probe VI-Fwd and LUC-1-Rev, which annealed within the intron sequence and the LUC coding region, respectively, were selected to measure only the unspliced luciferase transcripts. The graph shows the ratio of unspliced versus total luciferase RNA copies for both YY1 double mutant and wild-type reference construct, which was set equal to 1. The data are the means (±SE) of eight different experiments. Asterisk indicates statistical significance (t-test; *, p<0.05). (**B**) Effect of YY1 silencing on the splicing efficiency of the *UbC* intron. The absolute quantification assay described in **A** was performed on cDNAs obtained from HeLa cells cotransfected with P3 or YY1mut e–f reporter vector and YY1-specific or nonsilencing control siRNA, as indicated. Analysis was performed at 72 h post-siRNA transfection and results are expressed relative to the value obtained for the control siRNA sample, set as 1. The graph displays the means (±SE) of five different experiments. Asterisk indicates statistical significance (t-test; *, p<0.05). (**C**) Gel image showing the results of quantitative PCR displayed in **B** (lanes 1–4). P3 and P7 derived amplicons (lanes 5, 6) served as a reference for the unspliced or spliced transcripts, respectively. M, DNA molecular weight marker (lane 7) (**D**) Effect of splicing impairment on luciferase RNA decay. HeLa cells transfected with P3 or YY1mut e–f reporter construct were treated, 48 h post-transfection, with 5 µM actinomycin D. At the time points indicated, total RNA was extracted and analyzed by RealTime RT-PCR with the luciferase primer pair referred to above. Data, normalized to B2M, are expressed as a percentage of the time zero value detected for P3 or YY1mut e–f, respectively. P3, filled diamonds; YY1mut e–f, open squares. The graph shows the results of a typical RNA decay analysis. Similar results were obtained in three separate experiments. (**E**) Analysis of YY1 binding to *UbC* RNA by RIP, in HeLa cells. Immunoprecipitation with YY1 antibody or IgG (performed as described under Materials and Methods) was followed by qRT-PCR for *UbC* or the control RNA (18S rRNA). *Upper panel*, Etidium Bromide-stained gel. RT-PCR samples were loaded, as indicated. *Lower panel*, RT-PCR quantification of the indicated *UbC* fragments (exon 1 and 3′-UTR) and the 18S rRNA as a control. Data are shown as percent input and are the average±SE of six independent experiments (***, p<0.001).

In an attempt to reconcile all the experimental evidences, we searched for a possible link between YY1 consensus motifs and splicing, by investigating if mutations of intronic YY1 binding sequences could produce any effect on the splicing of the *UbC* intron. The reporter construct YY1mut e–f and the reference construct P3 were transiently transfected in HeLa cells. Splicing efficiency was investigated by reverse-transcription quantitative PCR assay on DNase-treated total RNAs. Two different primer pairs were used: LUC-Fwd and LUC-Rev, specific for the luciferase coding sequence, to amplify the total reporter transcripts (spliced and unspliced); intron probe VI-Fwd, complementary to an internal intron sequence, and LUC-1-Rev, matching to the 5′-luciferase coding region, to quantify only the intron-bearing luciferase RNAs (unspliced). To detect both mature and pre-mRNA, reverse transcription was performed with random hexamers as primers. An absolute quantification assay was developed with the aim to obtain more accurate data. Results, expressed as percentage of unspliced versus total luciferase RNA copies, revealed a fraction of unspliced transcripts ∼1.8-fold higher in YY1mut e–f reporter vector compared to the reference P3 (set equal to 1) (Figure 9A). Statistical analysis (t-test) showed that the difference was significant (p<0.05).

We next sought to investigate if, besides YY1 binding motifs, the efficiency of intron removal also depended on the presence and/or activity of the cognate *trans*-acting factor YY1. To address this point, we evaluated the splicing efficiency of the luciferase transcripts upon YY1 knockdown in HeLa cells, cotransfected with the wild-type P3 or the YY1mut e–f reporter construct. 72 h after siRNA delivery cells were processed to measure the efficiency of *UbC* intron splicing. Figure 9B shows that, in P3 cotransfected cells, YY1 silencing was accompanied by a 1.5-fold increase of the percentage of unspliced luciferase RNA, respect to the value detected in cells challenged with nonsilencing control siRNA. The difference was statistically significant (p<0.05). Reduction of YY1 protein levels did not further reduce the basically lower splicing efficiency of the YY1mut e–f construct (Figure 9B).

Products obtained by RT-PCR amplification (with primers flanking the intron) of RNAs extracted from HeLa cells transfected as in Figure 9B, are represented in Figure 9C. The gel image confirms the presence of the expected spliced and unspliced LUC RNAs and clearly shows the increase of the unspliced luciferase transcript variant upon mutagenesis of YY1 binding motifs and/or silencing of YY1 protein. Plasmids P3 and P7 were amplified as a control for the unspliced and spliced products, respectively.

To investigate if impaired splicing could affect RNA stability, cells transfected with wild-type P3 or the YY1mut e–f construct were challenged with actinomycin D to inhibit transcription. At the indicated time points, RNA was extracted and used to determine the decay rates of LUC transcripts. Luciferase mRNAs from both P3 and YY1mut e–f transfected cells exhibited half-lives of ∼5 h, indicating no significant differences in mRNA stability dependent on decreased splicing efficiency (Figure 9D).

To assess if the reduction of splicing efficiency of P3-driven transcripts following YY1 knockdown is specific to the *UbC* intron, we cotransfected YY1 silenced HeLa cells with a reporter construct similar to P3, harboring a chimeric intron in place of the *UbC* intron [19]. The copy number of unspliced luciferase transcripts (detected by the absolute quantification assay) was not statistically different in cells treated with YY1 siRNA (181±56; n = 3) or control siRNA (184±64; n = 3).

To confirm the impact of splicing on *UbC* regulation and to assess the role of YY1 in this event, we investigated intron retention by the endogenous *UbC* RNA upon YY1 silencing. YY1 knockdown significantly raised the amount of the unspliced endogenous *UbC* RNA (1.48±0.23-fold change vs control siRNA; p<0.05, n = 6) (Figure 9E). Thus, the reduced splicing efficiency upon YY1 silencing is specific to the *UbC* intron.

To get more insights about the mechanisms by which YY1 promotes intron splicing, we looked for possible YY1/RNA interactions *in vivo*. We performed RNA immunoprecipitation (RIP) with YY1 antibody, following formaldehyde-based crosslinking of RNA to proteins in HeLa cells. qRT-PCR of YY1 pulldown material showed significant coimmunoprecipitation of *UbC* RNA, as assessed with primer pairs specific for the exon 1 and the 3′-UTR of ubiquitin C RNA. A very low signal was detected with primers encompassing the YY1-e site in the intron region. The interaction was not detected in RT-negative samples and when IgG antibodies were used. Graph in figure 9F shows the percent input values for the exon 1 and 3′-UTR ubiquitin target regions. At these qPCR positions, *UbC* pulldown by anti-YY1 was enriched above background (i.e. the IgG control). The exon 1 domain and the 3′-UTR showed a 6.2- and 5.6-fold enrichment in the YY1 IP sample over the IgG control, respectively (p<0.001, for both *UbC* RNA domains). In the same experimental conditions, the very abundant 18S rRNA showed a lower percent input, with a not statistically significant fold-enrichment of YY1 over the IgG sample. Results are the means (±SE) of six independent experiments. The gel shows that both specific and control primers amplified the expected fragments from the input sample. However, they did not produce a detectable signal from control RIP performed with IgG antibodies or from the no-RT controls.

## Discussion

The broad application of mammalian ubiquitin C promoter in vectors for gene delivery to direct constitutive high levels of transgene expression, is not balanced by a great deal of data on the molecular mechanisms regulating promoter activity [9], [10], [37]. *UbC* gene represents an essential source of ubiquitin during episodes of stress and is also required to meet the physiological demand of Ub for cellular function and survival [7], [17]. By targeted disruption of the gene, Ryu and coauthors demonstrated that *UbC* function cannot be compensated by other ubiquitin genes [7]. The regulatory mechanisms underlying *UbC* gene transcription remain, up to date, unclear.

Our previous work revealed that the unique 5′-UTR intron of the *UbC* gene is required for the maximal activity directed by the proximal promoter sequence [19] and that Sp1 and YY1 transcription factors are able to interact, *in vitro*, with multiple binding sites within most part of the intron region. In the current study we identified four Sp1 and two YY1 binding sites within the intron sequence.

By site-directed mutagenesis, we demonstrated that abrogation of Sp1 binding sites, one at a time, does not affect reporter expression. The same result was obtained when all the Sp1 binding sites were mutagenized in the same construct, excluding the possibility that the redundant sites are alternatively used to transactivate the promoter. On the whole, these data rule out the participation of the Sp1 protein to the enhancer behavior of the intron, although they do not exclude a role for Sp1 in the regulation of *UbC* expression. Indeed, Marinovic et al. [38] demonstrated the involvement of Sp1 in ubiquitin C induction by glucocorticoids, by interaction with a binding site comprised in the proximal promoter region cloned in P3. ChIPseq datasets for Sp1 protein generated in different mammalian cell lines showed a peak of Sp1 binding in the proximal promoter and no signal within the intron sequence (http://www.ncbi.nlm.nih.gov/geo; accession record GSM803363), thus supporting both published data [38] and our findings.

To investigate the role of YY1 transcription factor, we generated three constructs carrying mutations in the proximal, distal, or both YY1 binding sites, respectively. Mutagenesis of the most 5′ YY1 binding site caused a significant reduction of promoter activity (by ∼55%), while the distal site mutation only slightly reduced reporter expression (by around 15%), and the construct carrying nucleotide changes in both YY1 binding motifs exhibited the greatest drop in luciferase expression, arguing an additive effect of the two DNA-binding sites. Moreover, using *in vitro* and *in vivo* studies, we confirmed that YY1 binds to this intron region, and by plasmid ChIP assay we demonstrated that it specifically requires the ATGGCGG motifs for intron recognition.

In support to our findings, the ChIPseq datasets for YY1 protein generated in different mammalian cell lines clearly showed a prominent peak of YY1 binding to the *UbC* intron sequence. Datasets are available for download from NCBI’s Gene Expression Omnibus (GEO) repository (http://www.ncbi.nlm.nih.gov/geo) under the accession records: GSM803406; GSM803513; GSM803381; GSM803535). When aligned with published ChIPseq datasets [39], the most 5′ intronic YY1 binding site, characterized in HeLa cells, exhibits a consistent overlap. Therefore, YY1 might actually be the molecular factor responsible for most of the boost of gene expression measured when intron is included in the reporter constructs.

The Gli-Kruppel-type transcription factor Yin Yang 1 (YY1) is a ubiquitously expressed, multifunctional protein that can function as an activator, repressor, or initiator binding protein depending on promoter context, chromatin structure, and interacting proteins [40]. YY1 has been implicated in the regulation of a large number of mammalian genes [41]. Moreover, YY1 was also found to affect gene expression by interaction with binding motifs positioned downstream of the transcriptional start site, within the coding region [42] or the intron sequences of target genes [43], [44].

In this study, we have functionally characterized the role of YY1 in the intron-dependent transactivation of the *UbC* gene. Overexpression of YY1 in HeLa cells did not increase either endogenous ubiquitin *C* or reporter luciferase expression, at least in the time window of transient transfection. However, it is possible that the significantly increased YY1 levels in this cervical carcinoma cell line [34] impede a further raise in target gene expression upon ectopic transfection of the factor, as also reported by others [35]. Knockdown of YY1 caused *UbC* expression levels to fall by approximately 15 and 23% at 48 and 72 h, respectively. A longer time-course of siRNA delivery was not possible as the cells could not tolerate YY1 reduction and began to die [31]. Knockdown of YY1 significantly decreased luciferase expression (by ∼30%, at 72 h post-siRNA delivery) driven by the construct P3, which bears wild-type intronic YY1 binding sites. No significant decrease was detected when the construct mutagenized in both YY1 binding motifs was cotransfected. These data indicate that the drop of luciferase expression upon knockdown of endogenous YY1 protein is mainly related to the presence of intact intronic YY1 binding sites.

To shed more light on the mechanism of YY1-mediated activation of *UbC* promoter, we investigated whether it was able to positively affect gene expression in a position- and orientation-independent fashion, as typical enhancers do [13]. Placing the *UbC* intron, alone or with the flanking exon 1, upstream of the proximal promoter, in both sense and antisense orientation, caused a drastic drop of luciferase expression, demonstrating that the intron was not able to support expression when localized outside the transcribed region, even if the other promoter elements were present. This evidence suggests that the *UbC* intron is not acting as a typical transcriptional enhancer, but rather regulates gene expression in a context-dependent manner, as reported for other intronic *cis*-elements which affect promoter activity orientation- or orientation- and position-dependently [45], [46].

As an alternative, we have hypothesized that the *UbC* intron could activate the so-called intron-mediated enhancement (IME), which is a rather ill defined process, largely described in plants, where it was found to be a much more common event than initially suspected, involving several different genes, including polyubiquitin coding genes [12], [13], [47]. To our knowledge, very few examples of IME outside the plant world have been documented [48]–[50]. IME is not due to the presence of enhancers within the intron sequence, although they may be present. Differently from typical enhancers, which may be located in whatever position and orientation with respect to the transcription start site, introns acting by IME must be located within transcribed sequence in order to boost gene expression [13], exactly as for the *UbC* intron.

One controversial point related to IME is its relationship with splicing. While splicing *per se* is not sufficient to account for IME, as argued from the evidence that not all spliced introns elicit an IME mechanism, yet splicing of enhancing introns seems to be an essential event to achieve maximal enhancement [13], [14], [36], [47]; however, unspliceable mutants able to induce mRNA accumulation almost as the wild-type intron bearing construct were also described [51].

When we tested the effects of mutations in the splice recognition sites of the *UbC* intron in our transient expression system, we found that the splicing defective reporter construct could not enhance luciferase expression at all, at either protein activity or mRNA level. Thus, we cautiously concluded that splicing of *UbC* intron is part of the mechanism that sustains IME. However, the retained intron might have caused Nonsense-Mediated Decay (NMD), due to internal ATG and in frame termination codons. We are aware that to cleanly evaluate the effect of intron splicing abrogation on the enhancement, it would be necessary to eliminate the ability of the unspliced intron to cause NMD [36]: however we didn’t introduce point mutations to remove internal start codons, since two ATG triplets overlap with the core region of YY1 binding motifs, making thus impossible to dissect the mere role of splicing from the effects mediated by internal YY1 consensus sequences.

As stated above, IME is a still largely unknown mechanism and attempts to identify sequences responsible for IME have had limited success; available data only refer to plant genomes, for which an algorithm, called IMEter, was recently developed to predict how much an intron enhances gene expression, without direct testing [12], [47]. However, up to date, no information is available about intron sequences possibly involved in IME activity in mammals.

Results obtained in this study demonstrate that both intact consensus splice sites and YY1 transcription factor binding sites are important for the intron to retain the maximal enhancing properties. This evidence prompted us to investigate if mutagenesis of YY1 binding sequences could affect splicing efficiency: an increased fraction of unspliced luciferase transcripts was, indeed, detected upon mutagenesis of both YY1 target sequences. Inferring splicing efficiency from the pre-mRNA/total RNA ratio does not allow to take into account the different stability of the two forms, however, in our opinion this means that the percentage of unspliced RNA could actually be even higher. On the other hand, luciferase RNA decay rates were similar in P3 and YY1mut e–f transfected cells (half-life of ∼5 h), indicating no significant difference in mRNA stability dependent on partial splicing inhibition [52].

Coming back to the outcome of YY1 mutant sites, we sought to determine if these intronic sequences were among the known intronic splicing regulatory elements (ISREs) [53]. Bioinformatic analysis of the intron sequence with RegRNA (http://regrna.mbc.nctu.edu.tw/) revealed that the consensus motif of the splicing factor Nova-1 (YCAY) is contained within the core YY1 binding site; thus it may be easily hypothesized that mutagenesis of YY1 target sequences simultaneously destroy two Nova-1 binding sites, causing impairment of splicing, although many more Nova-1 sites are present within the intron spanning sequence. Moreover, Nova-1 has been reported to mostly regulate neuron-specific alternative splicing [54].

However, we remind that transcription factor YY1 does play an active role in the intron-dependent increase of reporter gene transcription, as argued from knockdown experiments. To explore whether the YY1 effect on gene expression was related, in some way, to intron splicing, we measured the splicing efficiency of luciferase RNA in cells receiving YY1 specific siRNA. A statistically significant increase of unspliced luciferase transcripts was observed upon YY1 silencing. The same effect was detected for the endogenous *UbC* RNA. On the whole, these data indicate that both intact YY1 binding sequences and adequate YY1 intracellular levels contribute to the splicing of the *UbC* intron, which is essential for the intron-mediated enhancement.

A critical issue is how YY1 deals in this role: several possible mechanisms could be proposed and/or indirect effects cannot be ruled out.

Despite the lack of evidence in the literature for a potential role of YY1 in splicing, the presence of YY1 as a constituent of messenger ribonucleoprotein complexes (mRNPs) [55] and colocalization of the transcription factor with the spliceosomal protein ZNF265 [56] have been reported. Moreover, the documented RNA-binding ability of YY1 [57] supports the possible direct participation of YY1 in the splicing event.

To look for possible direct YY1/*UbC* RNA interactions *in vivo*, we performed RNA immunoprecipitation. A significant enrichment of the *UbC* RNA sequence was detected in the anti-YY1 RIPed sample, with primers targeting the exon 1 (which represents the 5′-UTR of the gene) or the 3′-UTR domain, while the signal from q-PCR of the intron domain was only barely detectable. This is rather expected since intron bearing precursor RNAs are promptly spliced out, mainly in the co-transcriptional phase [52]. Moreover, the exon 2 coding sequence of the *UbC* gene cannot be amplified because of the homology with the other ubiquitin genes [5].

On the whole, results of RIP assay indicate that *UbC* RNA makes direct contacts with YY1 *in vivo*, as revealed by qPCR of 5′-UTR (exon 1) and 3′-UTR positions. Although YY1 binds the ATGGCGG motif on intron DNA, its interaction with *UbC* RNA seems to occur through a different binding sequence, as also reported by others [57]. This dual role for YY1, which acts as both DNA and RNA binder can set the basis for an additional level of *UbC* gene regulation, enabling cross-talk between transcriptional/co-transcriptional pathways. Moreover, recent emerging literature highlights that extensive regions within 3′-UTRs of protein coding RNAs provide ample sequence elements that can be bound by proteins which function in the regulation of mRNA biogenesis, processing and distinct post-transcriptional processes [58].

Nevertheless, the above data do not exclude the coexistence of both direct and indirect mechanisms for YY1 participation in the *UbC* intron splicing. YY1 might directly bind *UbC* RNA to positively affect intron removal, as supported by RIP assay. It can also be hypothesized that YY1, bound to its target sequences within intron DNA, may serve as a docking protein for the spliceosomal complex or other splicing regulatory factors, improving their loading on the pre-mRNA substrate, thus indirectly promoting the splicing event. This would be in agreement with evidences that IME mostly acts at cotranscriptional level [12], [13].

Anyway, as stated by Parra et al. [12], “it is not yet known whether IME is mediated by DNA or RNA” and, to date, no evidence is available about the *trans*-acting molecular players able to interact with the enhancing signals. A more recent paper provides evidence for a DNA-based mechanism of IME, and suggests that intronic DNA structure or “factors” associated with DNA may, in some way, lead to a higher level of mature mRNA production [59]. These results weaken, but cannot entirely exclude, the hypothesis of IME operating at the RNA level, with the only firm point being that the exact mechanism underlying IME has yet to be determined.

Our data add a little piece of knowledge in this complex scenario. Indeed, we provide evidence that the intron-mediated enhancement of *UbC* gene expression requires a splicing-competent intron and the interaction of the sequence-specific DNA binding factor YY1 with its cognate *cis-*elements within the intron region. Moreover, YY1 motifs (i.e. the enhancing signals) and YY1 transcription factor (i.e. the trans-acting player) also affect the splicing efficiency, which we found to be essential for maximal enhancement. However, the effect of YY1 downregulation or the mutagenesis of its binding sites on intron retention is modest and does not account for the overall impact of YY1 on the *UbC* promoter activity, suggesting that most likely additional mechanism(s), like an atypical enhancer behavior and/or an IME-related effect, may be involved.

Evidences for a direct YY1/*UbC* RNA interaction *in vivo* were also provided.

More experimental work is needed to better dissect this complex topic. Anyway, our data suggest that the bivalent nature of YY1, binding both *UbC* RNA and intron DNA target sequences, might be at the basis of the molecular mechanism(s) involved.

Nevertheless, our findings are of great significance in view of the use of human *UbC* promoter in gene transfer applications [60]: uncovering the molecular mechanisms regulating *UbC* gene expression, may, in fact, have important implications for a more rational design of *UbC*-based vectors. *UbC* is already in the repertoire of currently employed promoters for its ability to drive robust transgene expression [37], [60], [61], but up to date the inclusion or not of the 5′-UTR intron in the *UbC*-based constructs has been performed on empirical basis [8], [9], [37]. The new information gained in our earlier [19] and current work could allow to exploit the intron-mediated enhancement as a method to further increase *UbC*-driven transgene expression. Additionally, dissecting the role(s) played by the extremely versatile YY1 transcription factor will, it is hoped, allow to fine-tune the regulatability of *UbC*-based expression vectors. Our data show that *UbC*-directed reporter expression is significantly impaired in YY1 silenced cells, thus highlighting, besides intron inclusion, a further layer of regulation of ubiquitin *C*-based gene transfer vectors, which depends on YY1 intracellular levels.
